# Anti-RNA polymerase III antibodies in systemic sclerosis: prevalence and clinical associations from a systematic review and meta-analysis

**DOI:** 10.1093/rheumatology/keaf392

**Published:** 2025-07-17

**Authors:** Abderrahmane Elhannani, Marie-Elise Martel, Aurore Collet, Aurélien Chepy, Sébastien Sanges, Éric Hachulla, Sylvain Dubucquoi, David Launay, Vincent Sobanski

**Affiliations:** Univ. Lille, Inserm, CHU Lille, U1286 – INFINITE – Institute for Translational Research in Inflammation, Lille, France; Département de Médecine interne et Immunologie Clinique, CHU Lille, Centre de Référence des Maladies Auto-immunes Systémiques Rares du Nord et Nord-Ouest, Méditerranée et Guadeloupe (CeRAINOM), Lille, France; Univ. Lille, Inserm, CHU Lille, U1286 – INFINITE – Institute for Translational Research in Inflammation, Lille, France; Département de Médecine interne et Immunologie Clinique, CHU Lille, Centre de Référence des Maladies Auto-immunes Systémiques Rares du Nord et Nord-Ouest, Méditerranée et Guadeloupe (CeRAINOM), Lille, France; Univ. Lille, Inserm, CHU Lille, U1286 – INFINITE – Institute for Translational Research in Inflammation, Lille, France; CHU Lille, Institut d’Immunologie, Lille, France; Univ. Lille, Inserm, CHU Lille, U1286 – INFINITE – Institute for Translational Research in Inflammation, Lille, France; Département de Médecine interne et Immunologie Clinique, CHU Lille, Centre de Référence des Maladies Auto-immunes Systémiques Rares du Nord et Nord-Ouest, Méditerranée et Guadeloupe (CeRAINOM), Lille, France; Univ. Lille, Inserm, CHU Lille, U1286 – INFINITE – Institute for Translational Research in Inflammation, Lille, France; Département de Médecine interne et Immunologie Clinique, CHU Lille, Centre de Référence des Maladies Auto-immunes Systémiques Rares du Nord et Nord-Ouest, Méditerranée et Guadeloupe (CeRAINOM), Lille, France; Univ. Lille, Inserm, CHU Lille, U1286 – INFINITE – Institute for Translational Research in Inflammation, Lille, France; Département de Médecine interne et Immunologie Clinique, CHU Lille, Centre de Référence des Maladies Auto-immunes Systémiques Rares du Nord et Nord-Ouest, Méditerranée et Guadeloupe (CeRAINOM), Lille, France; Univ. Lille, Inserm, CHU Lille, U1286 – INFINITE – Institute for Translational Research in Inflammation, Lille, France; CHU Lille, Institut d’Immunologie, Lille, France; Univ. Lille, Inserm, CHU Lille, U1286 – INFINITE – Institute for Translational Research in Inflammation, Lille, France; Département de Médecine interne et Immunologie Clinique, CHU Lille, Centre de Référence des Maladies Auto-immunes Systémiques Rares du Nord et Nord-Ouest, Méditerranée et Guadeloupe (CeRAINOM), Lille, France; Univ. Lille, Inserm, CHU Lille, U1286 – INFINITE – Institute for Translational Research in Inflammation, Lille, France; Département de Médecine interne et Immunologie Clinique, CHU Lille, Centre de Référence des Maladies Auto-immunes Systémiques Rares du Nord et Nord-Ouest, Méditerranée et Guadeloupe (CeRAINOM), Lille, France; Institut Universitaire de France (IUF), Paris, France

**Keywords:** SSc, autoantibodies, RNA-polymerase, prevalence, systematic review, meta-analysis

## Abstract

**Objectives:**

Anti-RNA polymerase III antibodies (ARA) are frequent in systemic sclerosis (SSc). However, the reported prevalence is variable among studies and some clinical associations are debated. We aimed (i) to update the recent data on overall ARA prevalence in SSc and heterogeneity between centres; and (ii) to describe their clinical associations.

**Methods:**

A systematic review of the literature available up to June 2024 was carried out in Pubmed and Embase. Meta-analyses were performed to assess the prevalence and clinical associations of ARA in SSc, combined with meta-regressions in case of heterogeneity to identify potential cofactors.

**Results:**

Ninety-three studies corresponding to a total of 23 038 SSc patients were included in the meta-analysis. In this population, the overall seroprevalence of ARA was 9% (95% CI: 8–10) with a high degree of heterogeneity (*I*^2^ = 88%, *P* < 0.001). ARA positivity was significantly associated with diffuse cutaneous subset (OR: 2.20 [1.91–2.53]), joint manifestations (OR: 1.29 [1.01–1.66]), gastric antral vascular ectasia (GAVE) (OR: 2.70 [1.52–4.81]), heart involvement (OR: 1.93 [1.18–3.18]), scleroderma renal crisis (OR: 7.82 [5.79–10.57]), interstitial lung disease (ILD) (OR: 1.10 [1.00–1.20]) and cancer (OR: 1.86 [1.33–2.59]).

**Conclusion:**

This study provides an overall seroprevalence of ARA of 9% [8–10] and confirms that SSc patients with ARA are at higher risk of severe skin extension and renal crisis. It also highlights a positive association with cancer, GAVE, heart, joint involvement and ILD. ARA positive SSc patients could therefore benefit from an appropriate screening of these potentially severe complications.

## Graphical abstract

**Figure:**
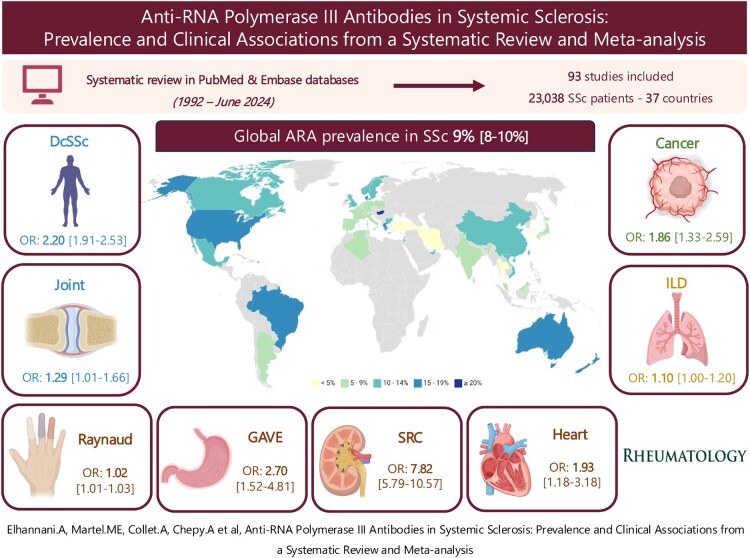

Rheumatology key messages

- Anti-RNA polymerase III antibodies (ARA) are present in 9% of scleroderma patients.
- ARA are associated to dcSSc, scleroderma renal crisis and cancer.
- ARA’s clinical phenotype also includes a higher risk of GAVE, heart involvement and ILD.

## Introduction

SSc is a heterogeneous autoimmune disease with a high morbidity and mortality [1, 2]. Antinuclear antibodies (ANA) are essential tools for clinicians involved in SSc patients care to better define the clinical phenotype and prognosis [3]. Anti-RNA polymerase III antibodies (ARA) were first identified in 1993 and are recognized as a highly specific biomarker for SSc [4], compared with antibodies reactive against RNA polymerase I or II described in other immune-mediated diseases [5, 6]. They are the third key autoantibodies encountered in SSc after anti-centromere antibodies (ACA) and anti-topoisomerase I (ATA) [1–3]. ARA have been included in the 2013 American College of Rheumatology/European League Against Rheumatism classification criteria (ACR/EULAR) for SSc [7]. Their estimated prevalence in a previous meta-analysis of 30 studies conducted by our team in 2014 was 11% (95% CI: 8–14) [8]. ARA assessment has become more common in routine clinical practice, leading to the evaluation of potentially new and different SSc populations using various detection methods. The prevalence is likely to have evolved since then. Association of ARA with some severe clinical manifestations of SSc is well known, such as diffuse cutaneous involvement (dcSSc) or scleroderma renal crisis (SRC) [9, 10]. More recently, ARA have been suggested to play a role in the immunological response to cancer [11–15], and have been reported as associated with synchronous cancers in patients with SSc [16]. Some authors have hypothesized that ARA could be associated with joint involvement [2, 17], silicone breast implants rupture [18–20] or gastric antral vascular ectasia (GAVE) [6, 21]. Beyond these well-known clinical associations, their relation to other key SSc features, like interstitial lung disease (ILD) or pulmonary hypertension (PH), remains undefined [3, 7, 22].

Therefore, this study aimed to (i) update the recent data on ARA overall prevalence in SSc, and (ii) describe their clinical associations through a systematic review and meta-analysis, accounting for potential cofactors.

## Materials and methods

### Systematic review and meta-analysis

The statement on Preferred Reporting Items for Systematic Reviews and Meta-Analyses (PRISMA) 2020 was used as a guide to conduct the review and analysis [23]. This study was declared on PROSPERO (n°CRD42024614350).

### Data sources and searches

Three of the authors (A.E., M.E.M. and V.S.) searched through published studies between January 1992 (the year preceding ARA first description) [4, 5] and June 2024, in PubMed and Embase databases. We used combinations of the terms ‘systemic sclerosis’, ‘scleroderma’, ‘anti-rna polymerase iii’, ‘dna-directed rna polymerases’ and ‘autoantibodies’. We adapted the search strategy to the specificities of each database. The reference lists of the retrieved papers were also searched to identify additional relevant publications.

### Study selection

Studies were included if they met the following criteria: (i) study data reported in English or French, (ii) patients over 18 years of age, (iii) with SSc diagnosis meeting the 2013 ACR/EULAR criteria [7] (or 1980 ACR criteria for previously published studies), (iv) with at least 30 patients with SSc tested for ARA.

Reports that did not provide sufficient information for data analysis were excluded. Two authors (M.E.M. and A.E.) independently reviewed the titles and abstracts of retrieved articles and applied the selection criteria to identify relevant material to be read in its entirety. Reviewers’ selections were compared, and, in case of disagreement, a third author (V.S.) was involved, and decisions were made by consensus. Reviewers independently read the full articles and applied the selection criteria to determine whether studies should be included in the meta-analysis. Selections were compared again, and a third author (V.S.) was involved in case of disagreement. Reports were included in the meta-analysis for each clinical association if data were available for both ARA positive and negative patients.

In addition, some of the studies initially selected contained overlapping cohorts for a given centre evaluated during the same period. We chose to include one study per centre (whichever study was the most recent and/or included the highest number of patients and/or for which clinical associations were best described). Studies comprising two or more cohorts were included if data extraction for each cohort was possible. In this case, each cohort was analysed as an independent cohort. Multicentre studies were excluded if the participating centres had published reports on a single cohort that was already included.

### Assessment of study quality and risk of bias

Two authors (M.E.M. and A.E.) independently assessed the quality of each study (risk of bias) using the Newcastle-Ottawa Scale (NOS) [24]. The items in each category were adapted as follows: in the selection field, the item ‘Is the case definition adequate?’ was replaced by ‘Is the autoantibody detection method well described and appropriate?’. In the item ‘Definition of Controls’, controls were SSc patients negative for ARA. In the comparability field, we assessed whether all patients had been tested for ARA. The item ‘Non response rate’ was omitted, giving a maximum of eight points per study.

### Data extraction

Relevant data were extracted from the selected studies using a standard form, including information on any clinical associations described in ARA positive and negative patients, and their definition criteria. After exclusion of clinical associations reported in less than five reports [25], the following elements were analysed: age at disease onset, duration of disease, sex ratio, Raynaud phenomenon, cutaneous form (percentage of dcSSc [26]), oesophageal involvement, gastrointestinal involvement, GAVE, muscle involvement, calcinosis, telangiectasia, digital ulcerations (DU), ILD, PH, cardiac involvement, SRC, history of cancer, overlap syndrome and primary biliary cirrhosis (PBC). The definition criteria for each parameter are detailed in Supplementary Data S1. Comparisons were made between ARA-positive and ARA-negative patients.

### Statistical analyses

Weighted and pooled summary estimates of the prevalence of ARA antibodies were calculated. For each meta-analysis, the *DerSimonian–Laird* method was used.

Consequently, studies were considered as a random sample from a population of studies. Odds ratios (ORs), adjusted for relevant clinical parameters of individual studies, were pooled using the random-effects model via generic inverse-variance weighting in both conventional and cumulative meta-analysis. Heterogeneity was quantified using a *χ*^2^ heterogeneity statistic and an *I*^2^ statistic for each analysis. The overall effect was estimated using a weighted average of the individual effects, with weights inversely proportional to the variance of the observed effects. The Freeman–Tukey transformation was used. Contour-enhanced funnel plots were checked to assess publication bias. Meta-regression was performed to assess the impact of autoantibody detection method, continent, country, age of cohort, female sex, disease duration, ILD, PH, DU, SRC, ACA and ATA. All statistical analyses were performed using R software, version 4.2.2; statistical significance was set at *P* < 0.05. For meta-analysis, the R package metafor was used.

## Results

### Studies included in the meta-analysis

**Figure 1.**
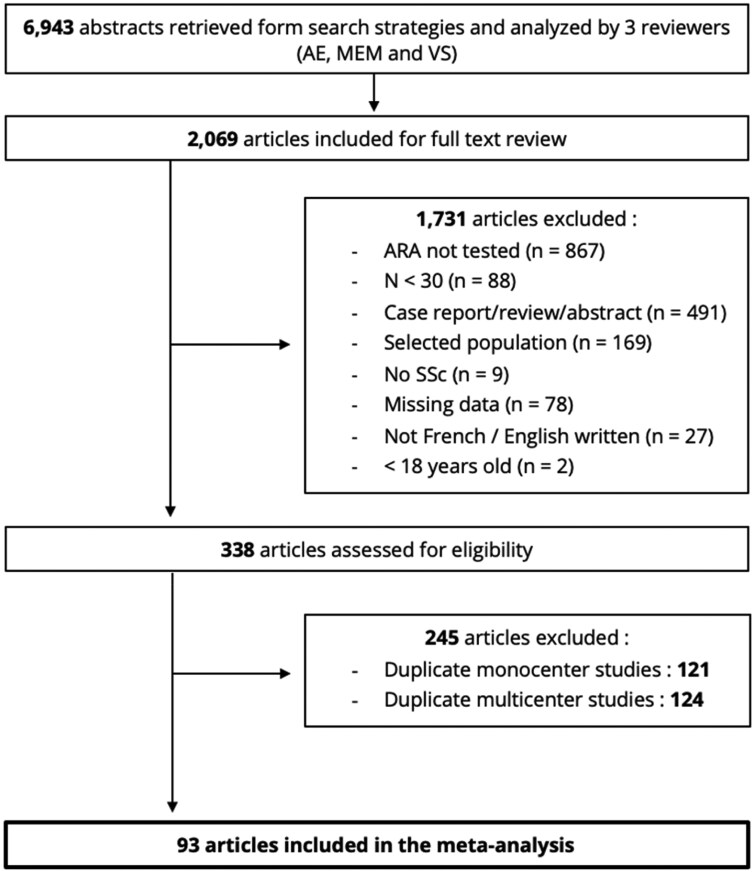
Flow chart illustrating the search strategy used to identify publications included in the meta-analysis. ARA: anti-RNA polymerase III antibodies

Using the aforementioned keywords, 6943 references were retrieved as a result of an electronic search of PubMed and Embase databases, of which 2069 articles were selected for full text review. Of these articles, 1731 were excluded (mainly because of patients untested for ARA, selected population or lack of clinical data in ARA positive and/or negative patients) and 338 articles were assessed for eligibility. A further 245 articles were excluded, mainly because of duplicate populations (Fig. 1). Ninety-three studies were eventually included in the meta-analysis (15, 27–118), representing a total of 23 038 SSc patients (2618 ARA-positive and 20 420 ARA-negative patients) from 37 different countries. In the whole population, 85% of the subjects were female (Supplementary Table S1). The modified version of the NOS score indicated that the included references were of good quality (Supplementary Fig. S1).

### Global prevalence of ARA

**Figure 2.**
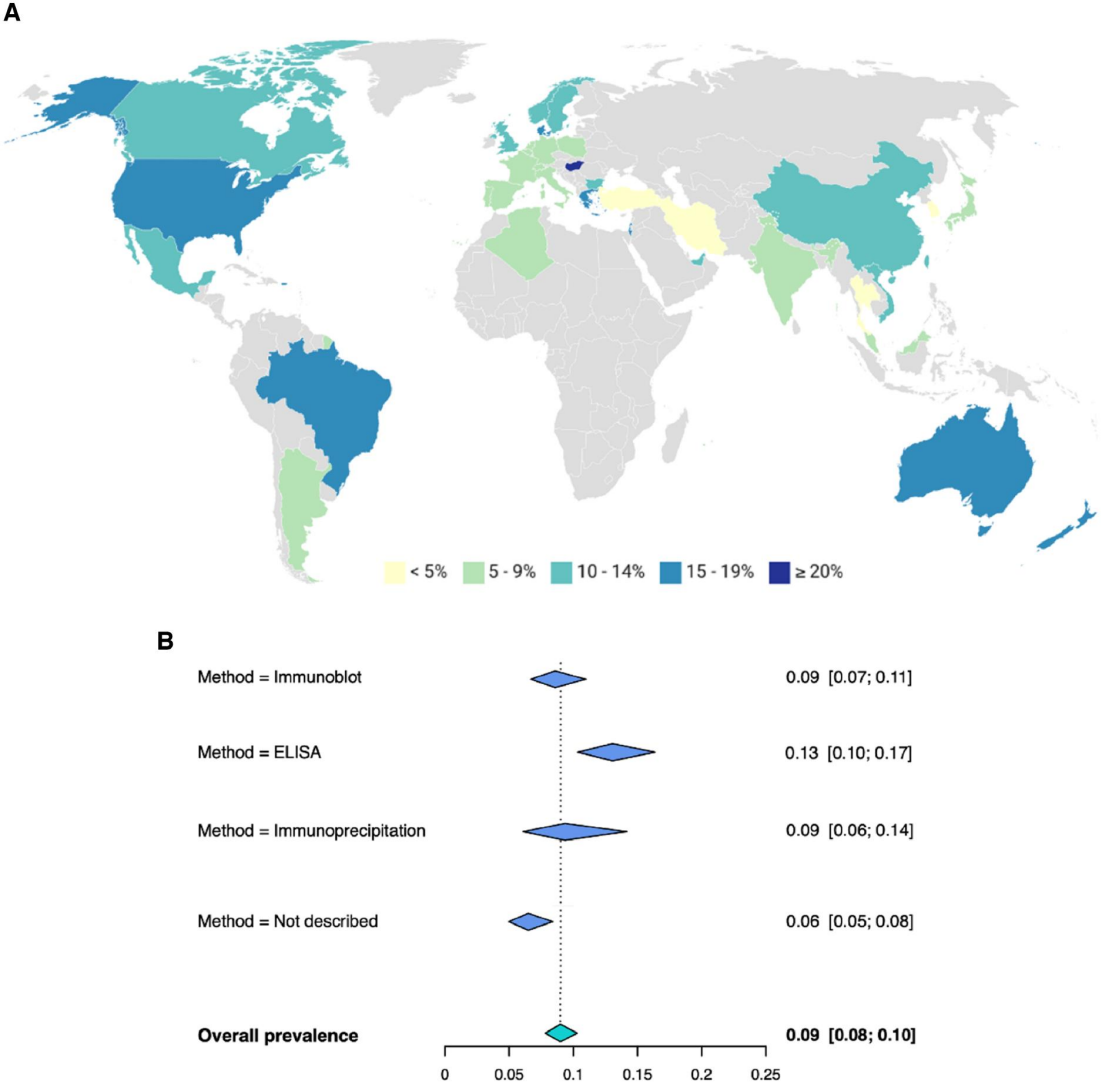
(**A**) Estimation of the worldwide prevalence of ARA in SSc according to countries of studies included in the meta-analysis. With the available data in the meta-analysis, the variable ‘country’ was associated with the prevalence of ARA. Grey areas = no data. (**B**) Prevalence of ARA in SSc patients, according to the method of detection. Diamonds represent the pooled prevalence for each detection method. ARA: anti-RNA polymerase III antibodies

ARA prevalence ranged from 1% to 41% and their overall pooled prevalence was 9% (95% CI: 8–10), with a high degree of heterogeneity (*I*^2^ = 88%, *P* < 0.001) (Fig. 2A and Supplementary Fig. S2). We assessed whether geographical factors, ARA testing methods or clinical characteristics of the study population available from the systematic review could explain this observed heterogeneity in ARA prevalence between studies. Meta-regression showed that the method of detection of ARA (*P* = 0.04), the country (*P* = 0.006), continent (*P* < 0.001) and the proportion of DU in the studied population (*P* = 0.01) were significantly associated with ARA prevalence. We could therefore presume an interaction between those specific population’s characteristics (reflecting inter-centres variability) and the estimated prevalence for each centre. However, the heterogeneity remained significant after inclusion of each of them individually in the model (Supplementary Table S2). After inclusion of all covariates in combination in a multiple meta-regression model, the residual heterogeneity was still statistically significant (Supplementary Table S3), suggesting that the observed variation in ARA prevalence is probably influenced by factors unreported in the included references.

Studies with ARA detected by ELISA showed a pooled prevalence of 13% (95% CI: 10–17) while studies using immunoprecipitation (IP) showed a pooled prevalence of 9% (95% CI: 6–14) and studies using immunoblot 9% (95% CI: 7–11) (Fig. 2B). Geographical areas of low ARA prevalence amongst SSc patients (1–13%) where mostly in Europe, whereas the geographical areas of the 22 studies with high ARA prevalence (15–27%) included Europe, North America, Asia (Israel, India and China) and two Australian studies (Fig. 2A).

### Clinical associations

**Figure 3.**
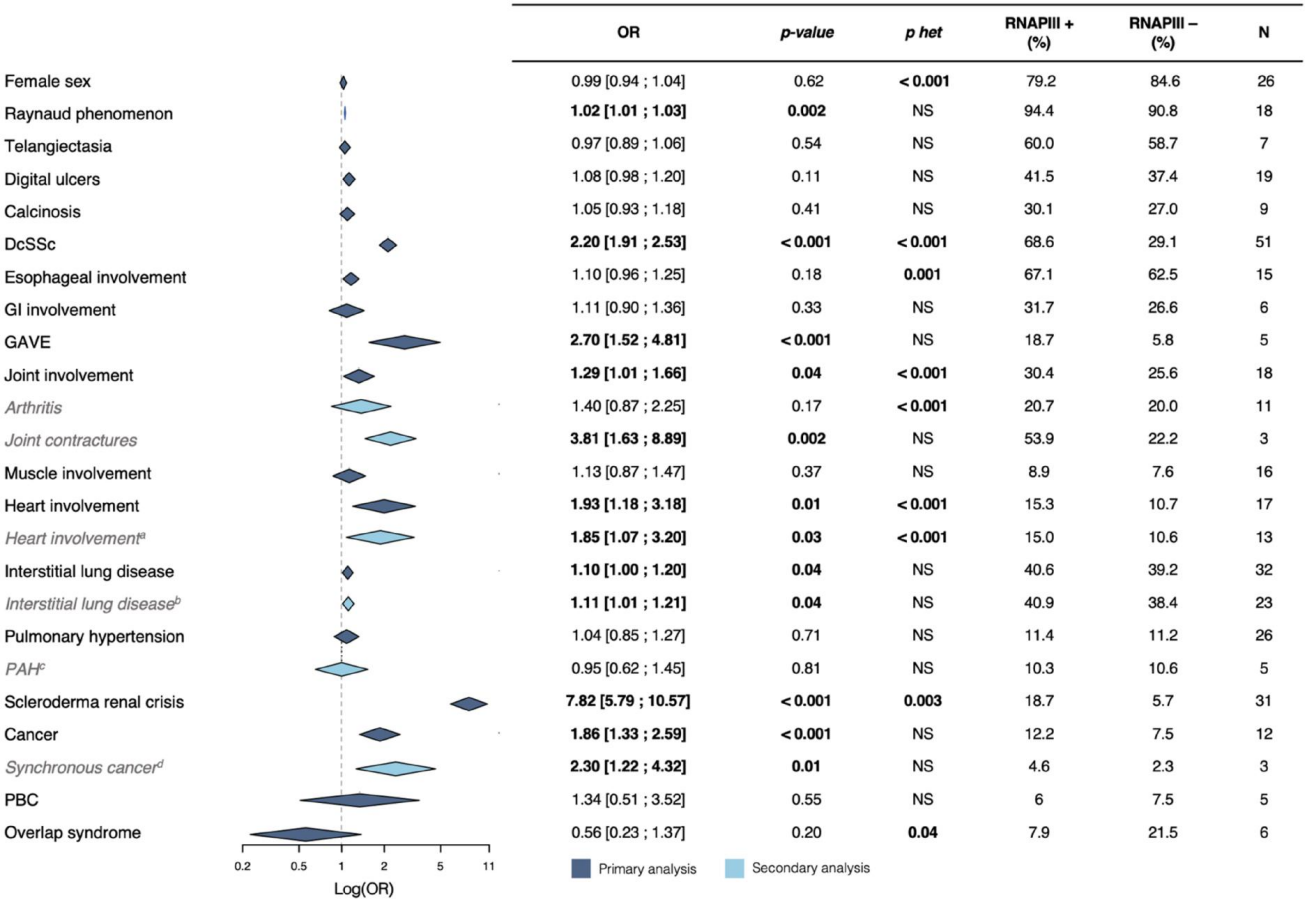
Association between ARA seropositivity and SSc features. Secondary analyses provided alternative odds ratio (OR) regarding: ^a^Cardiac involvement excluding studies in which the definition comprised right heart involvement or pericardial effusion, ^b^Interstitial lung disease diagnosed exclusively on high resolution computed tomography or chest X-ray, ^c^Pulmonary arterial hypertension (PAH) confirmed by right heart catheterization, ^d^Cancer diagnosed within five years before or after disease onset. *P* het: *P*-value for heterogeneity, NS: non-significant, ARA: anti-RNA polymerase III antibodies, GAVE: gastric antral vascular ectasia, GI involvement: gastrointestinal involvement, PBC: primary biliary cholangitis. Statistically significant associations are represented in bold

We collected all SSc manifestations available in the included references and then evaluated their association with ARA positivity. Fig. 3 summarizes the results of the different meta-analyses performed for each of these variables.

ARA patients had a mean age at disease onset (first non-Raynaud symptom) of 52.4 years vs 49.7 years (*P* = 0.04) in non-ARA patients (17 studies with available data). Their mean disease duration at inclusion in the study was about 5.7 years vs 7.4 years (*P* = 0.02) in non-ARA patients (16 studies with available data). ARA positivity was significantly associated with dcSSc (OR: 2.20 [1.91–2.53]), joint manifestations (OR: 1.29 [1.01–1.66]), GAVE (OR: 2.70 [1.52–4.81]), heart involvement (OR: 1.93 [1.18–3.18]), SRC (OR: 7.82 [5.79–10.57]), Raynaud’s phenomenon (OR: 1.02 [1.01–1.03]), ILD (OR: 1.10 [1.00–1.20]) and cancer (OR: 1.86 [1.33–2.59]).

No statistically significant association was found between ARA and female sex, PH, telangiectasia, history of DU, calcinosis, oesophageal involvement, gastrointestinal involvement, muscle involvement, overlap syndrome or primary biliary cirrhosis.

### Meta-regression analyses

Meta-regressions were performed to investigate the observed significant heterogeneity between studies when SSc manifestations and ARA were associated with a *P het* < 0.05, i.e. cutaneous subset, joint, heart involvement and SRC. It showed that the continent, disease duration, frequencies of ACA or ATA in the studies influenced the association between cutaneous subset and ARA seropositivity, with a significant residual heterogeneity (Table 1). After inclusion of those parameters, in combination, in the multiple meta-regression analysis, the residual heterogeneity was still statistically significant (Supplementary Table S4).

**Table 1.** Meta-regression analysis of the association between characteristics of included studies and ARA association to cutaneous subset, SRC, heart and joint involvement

Variables	Cutaneous subset		SRC		Heart		Joint	
	*P asso*	*P het*	*P asso*	*P het*	*P asso*	*P het*	*P asso*	*P het*
Publication date	0.09	NA	0.91	NA	**<0.001**	**0.19**	**<0.001**	**0.12**
Method of detection	0.34	NA	0.25	NA	0.54	NA	0.55	NA
Country	0.42	NA	0.60	NA	0.95	NA	**<0.001**	**0.52**
Continent	**0.02**	**<0.001**	0.72	NA	0.73	NA	0.97	NA
Age of cohort	0.31	NA	0.41	NA	**0.004**	**0.002**	0.46	NA
Female sex	0.31	NA	0.83	NA	0.92	NA	0.49	NA
DcSSc	–	–	0.74	NA	0.94	NA	0.84	NA
Disease duration	**0.05**	**<0.001**	0.99	NA	0.81	NA	0.77	NA
ILD	0.06	NA	0.92	NA	0.80	NA	0.87	NA
PH	0.85	NA	0.97	NA	0.10	NA	0.93	NA
Digital ulcerations	0.11	NA	0.32	NA	0.97	NA	0.29	NA
SRC	0.08	NA	–	–	0.72	NA	**0.02**	**0.006**
ACA	**0.003**	**<0.001**	0.73	NA	0.48	NA	0.53	NA
ATA	**<0.001**	**<0.001**	0.84	NA	0.95	NA	0.27	NA

NA: not applicable, ILD: interstitial lung disease, PH: pulmonary hypertension, SRC: scleroderma renal crisis, ACA: anti-centromere antibodies, ATA: anti-topoisomerase I antibodies, *P* asso: *P*-value for association, *P* het: *P*-value for heterogeneity. Statistically significant associations are represented in bold.

Publication date and country influenced the association between joint involvement and ARA positivity, with a non-significant residual heterogeneity (*P residual het *= 0.12 and 0.52, respectively) (Table 1). Indeed, the association between ARA and joint manifestations remained significant when considering articles published between 2010 and 2019 (OR: 1.52 [1.13–2.04] for studies published between 2010 and 2014; OR: 2.52 [1.74–3.63] for publications between 2015 and 2019), but not for previous nor later studies (Supplementary Fig. S3).

**Figure 4.**
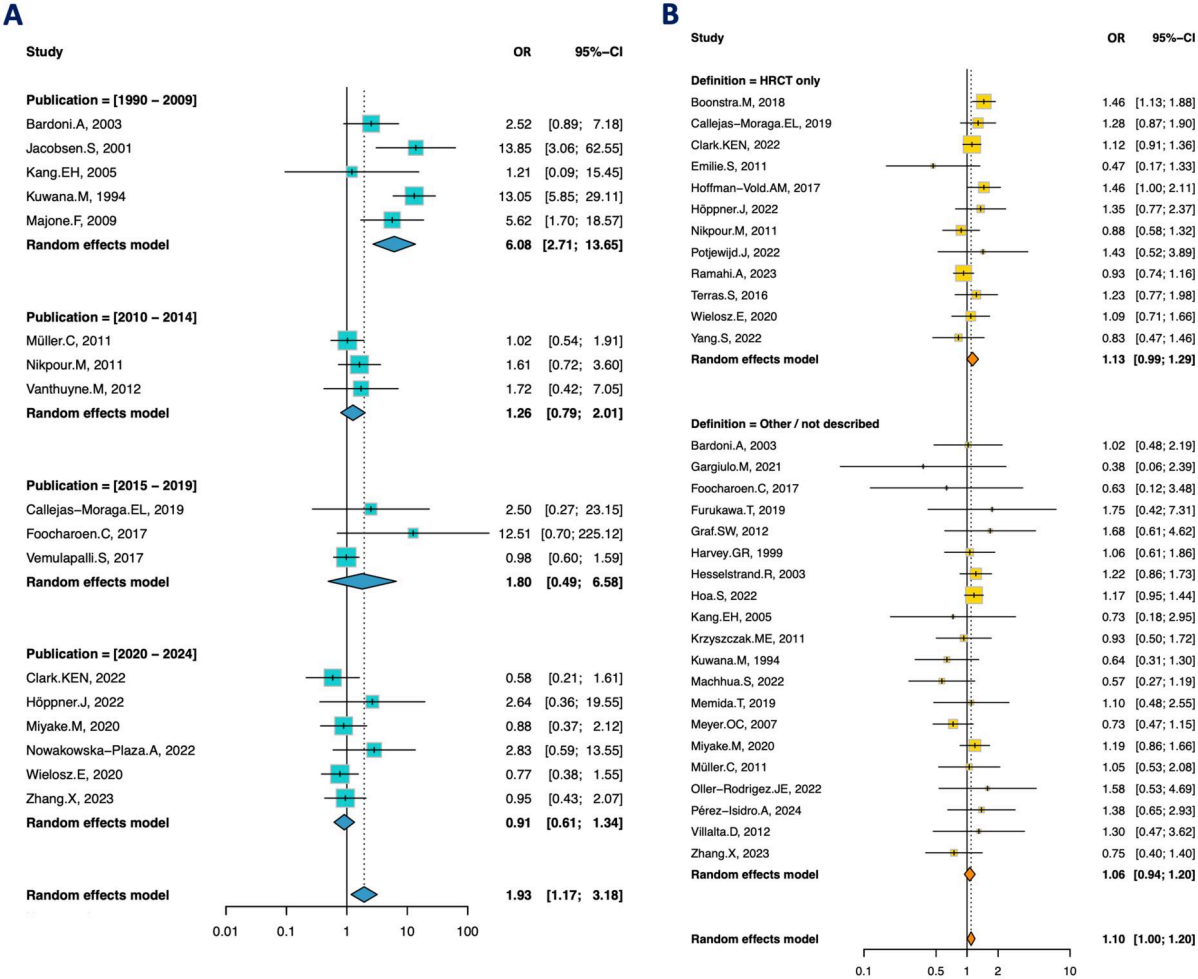
(**A**) Forest plot showing the association between heart involvement and ARA in SSc patients, in all studies, according to the publication year. Each square represents an individual odds ratio, with the size of the square being proportional to the weight given to the study. Lines represent the 95% confidence interval. Diamonds represent the pooled odds ratio for each publication time frame. (**B**) Forest plot showing the association between ILD and ARA in SSc patients, in all studies, according to the method used to define ILD. Each square represents an individual odds ratio, with the size of the square being proportional to the weight given to the study. Lines represent the 95% confidence interval. Diamonds represent the pooled prevalence for each diagnostic method. HRCT: high resolution CT

Publication date and mean age of patients included in each study influenced the association between heart involvement and ARA positivity (Table 1), with a significant residual heterogeneity for age. Articles published between 1990 and 2009 suggested that ARA was a strong risk factor for heart involvement (OR: 6.08 [2.71–13.65]), whereas when considering later published articles, the association remained positive but did not reach significance (Fig. 4A).

We did not find any factor, among the ones listed in Table 1, significantly associated with the relationship between SRC and ARA positivity.

### Secondary and sensitivity analyses

Considering the important heterogeneity amongst certain disease manifestation’s definition, secondary analyses with restricted definition criteria were performed to assess the consistency of our results and are presented in Fig. 3. The description of each refined organ involvement’s definition is available in Supplementary Data. In these secondary analyses, the association to heart involvement remained significant when excluding studies with an imprecise definition of primary heart involvement (PHI) (e.g. right heart failure or pericardial effusion alone) (OR: 1.85 [1.07–3.20]). Similarly, the association with cancer persisted when considering only synchronous cancer (OR: 2.30 [1.22–4.32]), although this information was only available in three out of the 12 studies included initially. Regarding joint involvement, when restricting the analysis to studies that specifically reported arthritis (i.e. excluding those that included arthralgia in the definition or did not specify the components of joint involvement) the association with ARA positivity was no longer significant (OR: 1.40 [0.87–2.25]). Joint contractures were associated with ARA (OR: 3.81 [1.63–8.89]) but only three studies provided relevant data. When only studies in which pulmonary arterial hypertension (PAH) confirmed by right heart catheterization (*n* = 5) were included, ARA positivity remained unassociated with PAH.

We then tested whether the criteria used to define ILD influenced its association to ARA. When considering studies that defined ILD based on radiological findings (on HRCT and/or chest X-ray) the association with ARA was significant (OR: 1.11 [1.01–1.21]) (Fig. 3). We identified 12 studies which used only HRCT for ILD definition. The association was no longer significant (OR: 1.13 [0.99–1.29]) (Fig. 4B).

**Figure 5.**
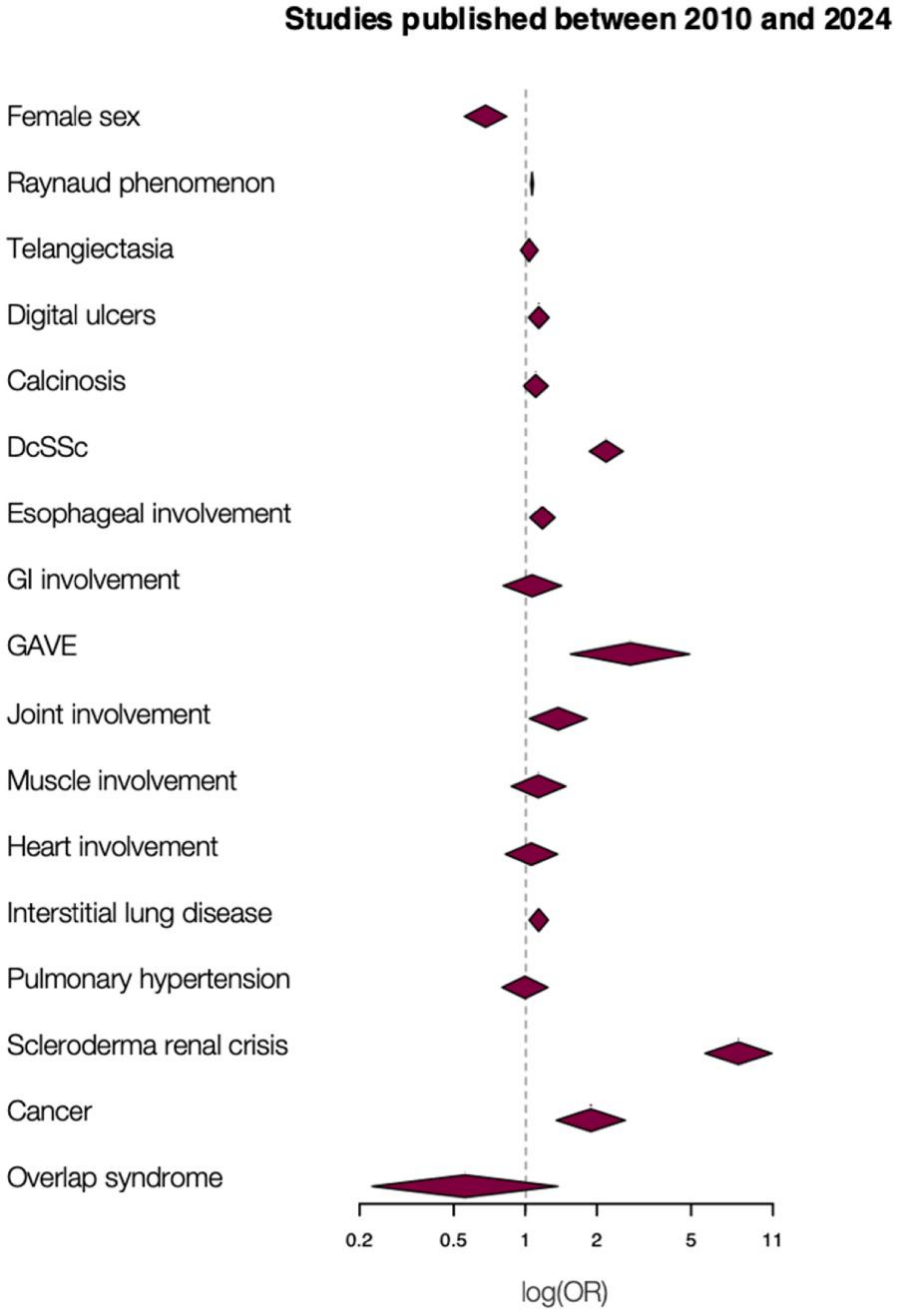
Sensitivity analysis of the association between ARA seropositivity and SSc features, using only studies published between 2010 and 2024. GI involvement: gastrointestinal involvement, GAVE: gastric antral vascular ectasia

Lastly, to assess the influence of the earliest studies in our results, we performed a sensitivity analysis by including only the studies published between 2010 and 2024. This analysis revealed a persistent positive association of ARA positivity with dcSSc (OR: 2.18 [1.85–2.56]), joint manifestations (OR: 1.36 [1.03–1.80]), GAVE (OR: 2.70 [1.52–4.81]), SRC (OR: 7.93 [5.74–10.95]), Raynaud’s phenomenon (OR: 1.02 [1.01–1.03]), ILD (OR: 1.12 [1.02–1.23]) and cancer (OR: 1.86 [1.33–2.59]). The main differences with the global meta-analysis were the negative association of ARA positivity with female sex (OR: 0.64 [0.53–0.79]) and the absence of significant association with heart involvement (OR: 1.05 [0.82–1.36]) (Fig. 5 and Supplementary Table S5).

## Discussion

The main results of this study can be summarized as follows: (1) the overall prevalence of ARA in SSc within the existing literature is 9% (95% CI: 8–10) but is variable, (2) meta-analyses confirmed associations of ARA with dcSSc, SRC, cancer and joint involvement, (3) they also highlighted that GAVE, ILD or heart involvement could be associated with ARA and (4) suspected negative or positive associations between ARA and other major clinical manifestations of SSc, like peripheral vascular manifestations (telangiectasias, DU), calcinosis, non-GAVE digestive involvement, muscular involvement or PH were not identified.

The estimated prevalence of ARA worldwide of 9% (95% CI: 8–10) was close to the results of a previous meta-analysis already carried out by our group in 2014 showing an overall prevalence of 11% (95% CI: 8–14) [8]. The first meta-analysis in 2014 was based on 30 studies and 8437 patients whereas this work includes 93 studies and 23 038 patients. It is therefore possible that populations with a naturally lower ARA prevalence have now been included. Moreover, ARA testing is now being more systematically implemented, the reported prevalence has likely adjusted to reflect their true frequency more accurately in diverse populations. Geographical parameters could explain partly the heterogeneity of ARA prevalence between centres and, to a lesser extent, some clinical associations. Firstly, genetic polymorphism (associated with ethnic origin) may influence clinical phenotypes and disease severity. GWAS-type studies have identified susceptibility loci within genes potentially involved in the regulation of inflammation or apoptosis [119–124]. Some HLA polymorphisms are linked with the occurrence of complications and the antibody profile [125–127]. As an example, HLA alleles DRB1*0407 and DRB1*1304 have been identified as risk factors for SRC, independently of clinical variables [128]. Secondly, occupational factors such as exposure to crystalline silica or organic solvents have been shown to play a role in the disease course [129, 130]. SSc mechanisms remain largely unknown, and other environmental factors such as bacterial or viral infections may also contribute to disease initiation [131]. None of these potential factors were available in the included studies, making it impossible to study their precise influence. Thirdly, heterogeneity between centres could be related to different clinical practices in assessing ARA (methods of detection) or screening for complications. Of note, the detection technique was not reported in 29% of articles (27/93). In studies comparing ELISA and IP for the diagnosis of SSc vs other autoimmune diseases, ELISA proved to be more sensitive and IP more specific [7, 39, 132, 133]. In this meta-analysis, ELISA tests accordingly resulted in a higher prevalence compared with IP or immunoblot in the overall population. In our 2014 meta-analysis, we suspected an association between ARA prevalence and detection methods, but the results were limited by the small amount of available data and did not reach significance [8]. This work, thanks to the number of studies included, revealed that this association was statistically significant. However, it only accounted for a small fraction of the total heterogeneity, meaning that its impact on the overall heterogeneity in ARA prevalence between studies is probably limited.

This meta-analysis suggested a positive association between heart involvement and ARA. Meta-regression showed that the date of publication of the study, and the age of the studied cohort could influence the association found between ARA and the occurrence of cardiac damage. Indeed, there was a strong clinical association for the five studies published between 1990 and 2009, with a still positive but less important clinical association for studies published between 2010 and 2014; 2015 and 2019. There was no significant association for studies published between 2020 and 2024. One of our hypotheses to explain these different results resides in a high degree of heterogeneity amongst studies regarding the definitions used for cardiac damage (EKG abnormalities, symptomatic heart failure or reduced left ventricular ejection fraction on cardiac ultrasound or MRI). In that regard, although several recent efforts [134, 135], notably illustrated in the EUSTAR group [136], have proposed comprehensive inclusion and exclusion criteria for PHI—such as incorporating multimodal imaging findings and ruling out confounding conditions like valvular disease, coronary artery disease, pulmonary hypertension—these have not yet been uniformly applied in the references included in our systematic review, contributing to the heterogeneity in our meta-analysis. To address this, we performed both primary and secondary analyses, including sensitivity analyses excluding studies that relied on isolated or non-specific criteria (e.g. right heart failure alone). Moving away from single-criterion definitions towards composite approaches is an important step forward. The World Scleroderma Foundation/Heart Failure Association definition [135] consensus represents a significant advance and will likely play a key role in standardizing definitions of PHI, thereby enabling more consistent and robust analyses in future studies.

Another intriguing result was the positive association between ILD and ARA. Several studies have suggested that ARA could be a protective biomarker for severe ILD [4, 10]. In a recent prospective observational study from Norway [58], 49% of 33 SSc patients with ARA had no ILD during a mean follow-up of 8 years. However, 18% of these patients with ARA developed extensive ILD on HRCT, with a later occurrence than in patients with ATA. In this work, although the association is more subtle and should therefore be interpreted with caution, our findings seem to indicate that ARA-positive patients may be at a slightly increased risk of ILD compared with other patients with SSc. A possible explanation for the heterogeneous findings regarding this association may lie in the variability of ILD’s definition criteria across studies. In the included reports, ILD was defined using various methods—ranging from HRCT and chest X-ray to combinations of clinical examination and pulmonary function tests. When the analysis was restricted to studies that defined ILD exclusively by HRCT or chest X-ray, the association with ARA remained significant. However, it became non-significant when we restricted the analysis to studies defining ILD exclusively by HRCT. Another key point to consider is that, due to limited data in the literature, we were unable to assess ILD severity, its progression over time or the influence of disease duration. It is possible that ARA-associated ILD presents distinct characteristics—potentially being less extensive, less progressive or with a later onset, than the forms typically encountered in patients with other serotypes like ATA. Therefore, further studies on the association of ARA with ILD, including precise lung features and degree of extension on HRCT, and their evolution over time, are still needed.

More recently, an increased risk of cancer has been suggested in ARA-positive patients. Several studies found a close temporal relationship between the onset of cancer and SSc in patients with ARA [4, 11–14], associated with a tumour RNA polymerase III expression in these patients [137]. Joseph *et al.* suggested that acquired mutations in the gene *RPC155/POLR3A* in tumours could trigger cellular immunity and cross-reactive humoral immune response. They hypothesized that acquired immunity could help to control naturally occurring cancers [13]. Breast and hematological malignancies seemed to be the most frequent cancers and were diagnosed within 5 years of SSc onset [11, 12, 14].

This work showed a higher risk of joint involvement in ARA patients. The frequency of joint involvement was significantly higher in ARA patients than in non-ARA patients in the EUSTAR registry (46% vs 30%, respectively) [15]. This result is complementary to the recent findings that joint involvement is associated with a more severe phenotype and disease progression [16].

Our meta-analysis has several limitations. This work was mostly conducted with retrospective studies, and non-standardized definition of each clinical feature in those studies. However, previously proposed definitions of SSc-cardiac or pulmonary disease have been improved over time and could lead to the identification of new clinical associations [135, 138]. Some studies did not specify the ARA detection method used. These limitations should be contrasted with the relatively good quality of the included studies according to the NOS scores. The number of studies and sample size varied considerably from one centre to another. There were also discrepancies between a country’s population size and the sample size available for analysis. Lastly, to avoid potential overlap from duplicate centres, we chose to include only monocenter studies or multicentre studies that also provided data for individual centres. As a result, some multicentre studies (e.g. the EUSTAR study of the association between malignancies and ARA [16]) had to be excluded due to the absence of this information, even though they might have offered complementary insights into certain clinical associations.

In conclusion, this meta-analysis provides an overall seroprevalence of ARA of 9% [8–10] and confirms that SSc patients with ARA are at higher risk of dcSSc, SRC and cancer. Merging results from numerous studies also highlighted ARA as a biomarker of joint involvement, GAVE, but also as a risk factor for heart involvement and ILD. Further studies are needed to confirm these results. Patients carrying ARA should therefore benefit from an appropriate screening of these potentially severe complications.

## Supplementary Material

keaf392_Supplementary_Data

## Data Availability

The data underlying this article are available in the article and in the supplementary files.
