# Supplementary material for: Anti-RNA polymerase III antibodies in systemic sclerosis: prevalence and clinical associations from a systematic review and meta-analysis
Source: Rheumatology (Oxford). 2025 Jul 17;64(12):6023–35. doi: 10.1093/rheumatology/keaf392 (PMC12671874; doi:10.1093/rheumatology/keaf392)
Supplement: keaf392_Supplementary_Data [file keaf392_supplementary_data.docx]

Definitions of SSc features

Depending on the study, the definition of clinical features could vary as follows:

- ILD could be defined by chest radiography and/or high-resolution computed tomography (HRCT) and/or velcro-type crackles on physical examination +/- alveolitis on bronchioloalveolar lavage +/- pulmonary function tests.
- PH could be defined as mean pulmonary artery pressure ≥ 25 mmHg by right heart catheterization (+/- pulmonary capillary wedge pressure ≤ 15 mmHg) and/or elevated estimated pulmonary artery systolic pressure on echocardiography.
- Joint involvement included arthritis and/or arthralgia.
- Skin involvement was based on the classification of Leroy *et al.*^1^
- Heart involvement could include: bundle branch blocks, arrhythmia, diastolic abnormalities, congestive heart failure, reduced left ventricular ejection fraction (LVEF), pericarditis, reduced diffusion capacity of lungs for carbon monoxide (DLCO) not attributable to ILD/PH, ischemic cardiopathy, echocardiographic abnormalities, EKG alterations without other causes, NT-pro-BNP and/or troponin abnormalities, evidence of myocardial fibrosis on heart biopsy or magnetic resonance imaging, reversible myocardial perfusion defects on thallium scintigraphy.
- Oesophageal involvement included oesophageal hypomobility, heartburn and dysphagia.
- Gastrointestinal involvement included early satiety, diarrhea, chronic pseudo-obstruction and constipation.
- Muscle involvement definition could rely on muscle pain and/or weakness +/- elevation in serum creatine kinase (CK) level, abnormalities on electromyogram (EMG) and/or magnetic resonance imaging (MRI); or muscle biopsy.
- SRC was defined as the onset of arterial hypertension/malignant hypertension associated with a rapid increase in serum creatinine level, with sometimes hemolytic anemia.
- PBC was diagnosed combining laboratory, immunological, and/or histological data according to the European Association for the Study of the Liver Clinical Practice Guidelines.
- Overlap syndromes were considered when patients exhibited features of two or more autoimmune rheumatic diseases (AARDs).

Definitions of SSc features in secondary analyses

- Heart involvement in the secondary analysis could include: bundle branch blocks, arrhythmia, diastolic abnormalities, reduced LVEF, symptomatic pericarditis, echocardiographic abnormalities, EKG alterations, NT-pro-BNP and/or troponin abnormalities, evidence of myocardial fibrosis on heart biopsy or magnetic resonance imaging, after excluding causes other than SSc.
- Interstitial lung disease diagnosed exclusively on high resolution computed tomography and/or chest X-ray.
- Pulmonary arterial hypertension was defined as mean pulmonary artery pressure ≥ 25 mmHg, pulmonary capillary wedge pressure ≤ 15 mmHg and peripheral vascular resistance ≥ 3 Wood Units on right heart catheterization.
- Synchronous cancer was defined as a cancer diagnosed within five years before or after disease onset.

| **Studies** | **Country** | **ARA**  **(n/N)** | **ARA**  **(%)** | **Detection method** | **Age**  **(years)** | **Female**  **(%)** | **DcSSc**  **(%)** | **ILD**  **(%)** | **PH**  **(%)** | **SRC**  **(%)** |
| --- | --- | --- | --- | --- | --- | --- | --- | --- | --- | --- |
| Abbot S, et al. 2020^2^ | Australia | 5/65 | 8 | Not described | 58.6 | 84.6 | 12.3 | 23.1 | 16.9 | 1.5 |
| Aguila LA, et al. 2021^3^ | Brazil | 20/298 | 7 | Immunoblot | 56.0 | 87.0 | 27.8 | 73.1 | 16.9 | 1.2 |
| Andréasson K, et al. 2014^4^ | Sweden | 15/302 | 5 | Not described | 52.1 | 85.8 | 17.5 | NA | NA | NA |
| Bardoni A, et al. 2003^5^ | Italy | 9/115 | 8 | Immunoprecipitation | 55.4 | 86.1 | 35.7 | 43.5 | 8.7 | 6.1 |
| Bass RD, et al. 2024^6^ | USA | 37/402 | 9 | Not described | 48.0 | 86.8 | 44.3 | 63.7 | 16.7 | 5.7 |
| Boonstra M, et al. 2018^7^ | Netherlands | 22/398 | 6 | CLIA | 55.0 | 81.3 | 23.6 | 53.6 | 5.9 | 3.9 |
| Bragazzi NL, et al. 2019^8^ | Israel | 248/1651 | 2 | ELISA | 62.7 | 81.7 | NA | NA | NA | NA |
| Caetano J, et al. 2022^9^ | Portugal | 14/95 | 1 | Not described | 51.9 | 86.3 | 35.8 | 37.9 | 10.5 | 2.1 |
| Callejas-Moraga EL, et al. 2019^10^ | Spain | 26/221 | 12 | Immunoblot / ELISA | 46.9 | 86.9 | 24.9 | 43.4 | 11.8 | 2.7 |
| Catano J, et al. 2019^11^ | France | 3/210 | 1 | Not described | 31.0 | 90.0 | 38.5 | 41.0 | 3.3 | 3.3 |
| Cavazzana I, et al. 2011^12^ | Italy | 14/144 | 10 | ELISA | 49.4 | 86.9 | 33.8 | 44.4 | 12.6 | 3.5 |
| Ceribelli A, et al. 2018^13^ | Italy | 1/71 | 1 | Immunoprecipitation | 66.0 | 95.3 | 16.9 | 32.4 | 26.8 | NA |
| Chang M, et al. 1998^14^ | USA | 9/89 | 10 | Immunoprecipitation | 60.2 | 76.4 | 40.5 | NA | NA | 2.2 |
| Clark KEN, et al. 2022^15^ | UK | 143/978 | 15 | ELISA | 65.7 | 83.7 | 35.0 | 40.8 | 11.4 | 6.8 |
| Coffey CM, et al. 2021^16^ | USA | 4/44 | 9 | Not described | 55.4 | 90.6 | 14.1 | 9.4 | 8.2 | NA |
| Dave J, et al. 2022^17^ | India | 3/60 | 5 | Immunoblot | 36.5 | 95.0 | 66.7 | NA | NA | NA |
| De Almeida Chaves S, et al. 2021^18^ | France | 16/375 | 4 | Not described | 55.3 | 77.9 | 16.8 | 25.4 | 4.8 | 2.4 |
| Didier K, et al. 2020^19^ | France | 2/34 | 6 | Immunoblot | 59.5 | 85.3 | 23.5 | 26.5 | 8.8 | 5.9 |
| Do HTT, et al. 2021^20^ | Vietnam | 7/57 | 12 | Immunoblot | 49.3 | 84.2 | 70.2 | 63.2 | NA | 5.3 |
| Emilie S, et al. 2011^21^ | France | 17/195 | 9 | ELISA | 48.5 | 83.6 | 36.9 | 35.9 | 13.3 | 6.7 |
| Foocharoen C, et al. 2017^22^ | Thailand | 4/285 | 1 | Immunoblot | 47.4 | 70.2 | 66.7 | 37.2 | 8.1 | 2.1 |
| Furukawa T, et al. 2019^23^ | Japan | 2/78 | 3 | Not described | 62.8 | 87.2 | 24.4 | 29.5 | 12.8 | NA |
| Gargiulo M, et al. 2022^24^ | Argentina | 8/135 | 6 | ELISA | 53.0 | 92.6 | 19.3 | 32.1 | 12.2 | 0.8 |
| Genrinho I, et al. 2023^25^ | Portugal | 1/102 | 1 | Not described | 57.0 | 82.4 | 29.4 | 21.6 | 8.8 | NA |
| Gindzienska-Sieskiewicz E, et al. 2019^26^ | Poland | 20/241 | 8 | Not described | 55.0 | 84.8 | 24.0 | 46.7 | 14.1 | 1.3 |
| González-Martín JJ, et al. 2020^27^ | Spain | 2/70 | 3 | Not described | 50.2 | 94.3 | 38.6 | NA | NA | NA |
| Graf SW, et al. 2012^28^ | Australia | 20/129 | 16 | Immunoblot | 42.8 | 85.2 | 27.9 | 13.2 | 15.0 | 3.9 |
| Günther F, et al. 2022^29^ | Germany | 3/49 | 6 | Immunoblot | 56.9 | 79.6 | NA | 51.0 | 10.2 | 4.1 |
| Harvey GR, et al. 1999^30^ | UK | 19/155 | 12 | Immunoprecipitation | NA | NA | 17.2 | 42.2 | NA | 15.6 |
| Hesselstrand R, et al. 2003^31^ | Sweden | 60/276 | 22 | ELISA | 49.2 | 74.0 | 24.6 | 36.2 | 20.6 | 2.2 |

| Hoa S, et al. 2022^32^ | Canada | 204/1698 | 12 | Immunoblot | 55.3 | 86.5 | 35.8 | 29.6 | 10.0 | 4.0 |
| --- | --- | --- | --- | --- | --- | --- | --- | --- | --- | --- |
| Hoffman-Vold AM, et al. 2017^33^ | Norway | 33/279 | 12 | Immunoblot | 49.0 | 78.9 | 27.2 | 37.3 | 18.3 | 3.6 |
| Höppner J, et al. 2022^34^ | Germany | 15/372 | 4 | Immunoblot | 48.0 | 82.8 | 28.0 | 34.9 | 12.4 | 2.7 |
| Horimoto AMC, et al. 2017^35^ | Brazil | 6/37 | 16 | ELISA | 50.6 | 96.6 | 27.0 | NA | NA | NA |
| Hübsch T, et al. 2023^36^ | France | 6/151 | 4 | Not described | 62.0 | 86.1 | 11.3 | 30.5 | 13.9 | 4.0 |
| Hysa E, et al. 2023^37^ | Italy | 24/601 | 4 | Not described | 57.6 | 92.8 | NA | NA | NA | NA |
| Jacobsen S, et al. 2001^38^ | Denmark | 39/174 | 22 | ELISA | 50.0 | 84.0 | 32.8 | 21.3 | NA | 1.7 |
| Jung S, et al. 2023^39^ | France | 4/39 | 10 | Not described | 51.7 | 89.7 | 46.1 | 23.1 | 5.1 | 0 |
| Kang EH, et al. 2005^40^ | Korea | 2/59 | 3 | Immunoprecipitation | 41.8 | 86.4 | 47.5 | 66.1 | NA | 1.7 |
| Karami J, et al. 2021^41^ | Iran | 9/504 | 2 | Not described | 41.3 | 86.0 | 59.0 | 47.4 | NA | NA |
| Korman BD, et al. 2017^42^ | USA | 54/198 | 27 | Not described | 52.6 | 83.3 | 41.4 | NA | 13.2 | NA |
| Korsholm SS, et al. 2022^43^ | Denmark | 4/102 | 4 | Not described | 59.5 | 76.5 | NA | NA | NA | NA |
| Krzyszczak ME, et al. 2011^44^ | USA | 17/105 | 16 | Immunoprecipitation | 50.6 | 84.8 | 54.3 | 43.8 | NA | NA |
| Kurteva E, et al. 2016^45^ | Bulgaria | 5/40 | 13 | Immunoblot | 50.0 | 90.0 | NA | NA | NA | NA |
| Kuwana M, et al. 1994^46^ | Japan | 14/275 | 5 | Immunoprecipitation | 41.7 | 88.7 | 25.8 | 52.8 | 6.2 | 4.0 |
| Lescoat A, et al. 2018^47^ | France | 6/103 | 6 | Not described | 57.9 | 74.8 | 34.0 | 45.6 | 5.8 | NA |
| Liaskos C, et al. 2017^48^ | Greece | 19/131 | 15 | Immunoblot | 58.1 | 84.7 | 37.4 | 32.5 | 15.3 | 1.3 |
| Lopez L, et al. 2023^49^ | France | 28/297 | 9 | ELISA | 62.4 | 73.5 | 31.0 | 35.9 | 6.5 | 3.5 |
| Low AHL, et al. 2012^50^ | Singapore | 3/68 | 4 | Immunoblot | 49.0 | 82.0 | 48.5 | NA | NA | NA |
| Machhua S, et al. 2022^51^ | India | 9/150 | 6 | Immunoblot | 40.3 | 92.0 | 50.0 | 76.0 | 20.6 | NA |
| Majone F, et al. 2009^52^ | Italy | 8/39 | 21 | ELISA | 50.9 | 97.4 | 59.0 | 61.5 | NA | 15.4 |
| Martel ME, et al. 2024^53^ | France | 11/300 | 4 | Immunoblot | 59.4 | 80.3 | 27.3 | 41.7 | 8.3 | 1.0 |
| Martins P, et al. 2021^54^ | Portugal | 11/251 | 4 | Not described | 59.0 | 87.6 | 25.9 | 33.9 | 10.4 | 2.0 |
| Memida T, et al. 2019^55^ | Japan | 3/41 | 7 | Not described | 62.8 | 85.4 | 34.1 | 71.4 | 13.9 | 15.8 |
| Meyer OC, et al. 2007^56^ | USA | 68/374 | 18 | Immunoprecipitation | 47.6 | 77.0 | 39.8 | 34.6 | 14.4 | 6.7 |
| Miyake M, et al. 2020^57^ | Japan | 52/546 | 10 | ELISA | 50.7 | 86.9 | 41.9 | 38.1 | 2.1 | 2.9 |
| Morozzi G, et al. 2011^58^ | Italy | 8/112 | 7 | ELISA | 56.3 | 88.4 | 42.0 | 65.2 | 39.3 | 1.8 |
| Motegi SI, et al. 2015^59^ | Japan | 14/246 | 6 | ELISA | 64.3 | NA | 31.7 | NA | NA | NA |
| Müller C, et al. 2011^60^ | Brazil | 35/85 | 4 | ELISA | 49.3 | 91.7 | 26.0 | 28.2 | 23.5 | 0 |
| Nagy G, et al. 2017^61^ | Hungary | 31/135 | 23 | Immunoblot | 49.0 | 83.4 | 40.8 | 53.8 | NA | 2.7 |
| Namas R, et al. 2023^62^ | United Arab Emirates | 14/108 | 13 | Not described | 45.6 | 86.8 | 54.5 | 53.3 | 17.4 | 0.6 |
| Nguyen B, et al. 2011^63^ | USA | 227/1191 | 19 | ELISA | 44.3 | 92.7 | 45.2 | NA | NA | 5.9 |

| Nikpour M, et al. 2011^64^ | Australia | 69/451 | 15 | ELISA | 58.1 | 88.0 | 29.3 | 30.8 | 10.9 | 5.4 |
| --- | --- | --- | --- | --- | --- | --- | --- | --- | --- | --- |
| Norman GL, et al. 2009^65^ | USA | 6/52 | 12 | ELISA | 54.8 | 84.6 | 36.5 | NA | NA | NA |
| Nowakowska-Plaza A, et al. 2022^66^ | Poland | 2/36 | 6 | Not described | 54.7 | 69.4 | 44.4 | 55.6 | 13.9 | NA |
| Oller-Rodrigez JE, et al. 2022^67^ | Spain | 4/42 | 10 | Not described | 59.2 | 95.2 | 47.6 | 33.3 | 11.9 | 4.8 |
| Osthoff M, et al. 2019^68^ | Switzerland | 17/211 | 8 | Not described | 60.0 | 79.0 | 18.0 | 49.0 | 15.0 | 2.0 |
| Pérez-Isidro A, et al. 2024^69^ | Spain | 5/97 | 5 | FEIA | 49.5 | 94.8 | 15.5 | 44.3 | 24.2 | 1.0 |
| Poormoghim H, et al. 2013^70^ | Iran | 10/100 | 10 | Immunoblot | 41.9 | 87.0 | 21.0 | 41.6 | NA | 6.0 |
| Potjewijd J, et al. 2022^71^ | Netherlands | 4/161 | 3 | Immunoblot | 56.5 | 73.3 | 13.7 | 36.6 | 20.5 | 2.5 |
| Ramahi A, et al. 2023^72^ | USA | 61/264 | 23 | ELISA | 53.1 | 78.1 | 45.9 | 64.5 | NA | NA |
| Rees MS, et al. 2021^73^ | New Zealand | 31/164 | 19 | Immunoblot | 63.0 | 86.6 | 25.6 | NA | NA | NA |
| Richardson C, et al. 2023^74^ | USA | 414/2379 | 17 | Immunoblot | 42.6 | 82.8 | 37.9 | 36.7 | NA | 4.5 |
| Serling-Boyd N, et al. 2020^75^ | USA | 26/100 | 26 | ELISA | 55.3 | 88.9 | 38.1 | 34.2 | 17.3 | 4.4 |
| Sheikh MB, et al. 2023^76^ | India | 6/30 | 20 | ELISA | 38.3 | 93.8 | 19.6 | 80.4 | 14.2 | NA |
| Smeets RL, et al. 2020^77^ | Netherlands | 15/346 | 4 | Immunoblot | 59.1 | 65.3 | 32.1 | 41.3 | 11.6 | NA |
| Sujau I, et al. 2015^78^ | Malaysia | 2/31 | 7 | Immunoblot | 51.3 | 90.3 | 29.0 | 71.0 | 58.1 | NA |
| Tahiat A, et al. 2020^79^ | Algeria | 9/150 | 6 | EIA | 45.4 | 93.3 | 28.0 | 70.0 | 10.7 | 0.7 |
| Tanaka H, et al. 2024^80^ | Japan | 11/112 | 10 | Not described | 67.3 | 90.1 | NA | NA | NA | NA |
| Terras S, et al. 2016^81^ | Germany | 11/158 | 7 | ELISA | 59.1 | 86.1 | 32.3 | 52.5 | 19.0 | 5.7 |
| Tsuji H, et al. 2022^82^ | Japan | 27/204 | 13 | ELISA / immunoprecipitation | 65.3 | 90.3 | 40.3 | 36.6 | 11.8 | 7.3 |
| Vanthuyne M, et al. 2012^83^ | Belgium | 23/319 | 7 | ELISA | 54.4 | 80.3 | 19.6 | 73.4 | NA | 3.7 |
| Vemulapalli S, et al. 2017^84^ | USA | 23/300 | 8 | Not described | 58.8 | 85.7 | 35.0 | 44.7 | 18.0 | NA |
| Villalta D, et al. 2012^85^ | Italy | 13/210 | 6 | Immunoblot | 58.6 | 92.4 | 30.5 | 19.5 | 13.8 | 1.4 |
| Watanabe T, et al. 2024^86^ | Japan | 14/156 | 9 | ELISA | 69.0 | 88.5 | 25.0 | 46.2 | 8.3 | 1.3 |
| Wielosz E, et al. 2020^87^ | Poland | 19/126 | 15 | Immunoblot | 53.8 | 77.8 | 47.6 | 54.0 | 22.2 | 7.1 |
| Yamaoka T, et al. 2008^88^ | Japan | 7/70 | 10 | ELISA / immunoprecipitation | 50.0 | 87.1 | 57.1 | 42.9 | NA | 3.4 |
| Yang S, et al. 2022^89^ | China | 11/64 | 17 | Immunoblot | 47.8 | 83.6 | 40.3 | 64.6 | 6.3 | 2.8 |
| Yayla ME, et al. 2018^90^ | Turkey | 2/93 | 2 | ELISA | 50.4 | 89.2 | 15.1 | 45.2 | 14.0 | NA |
| Yen TH, et al. 2023^91^ | Taiwan | 21/208 | 10 | LIA | 63.0 | 76.9 | 38.5 | 35.6 | 8.7 | 2.4 |
| Zebryk P, et al. 2023^92^ | Poland | 6/96 | 6 | Immunoblot | 53.7 | 85.0 | 33.0 | 69.0 | 13.0 | 1.0 |
| Zhang X, et al. 2023^93^ | China | 10/140 | 7 | Immunoblot | 54.2 | 89.3 | 50.7 | 65.7 | 16.4 | 2.1 |
| Ziswiler HR, et al. 2007^94^ | Switzerland | 2/33 | 6 | Not described | 57.5 | 84.8 | 21.2 | NA | NA | NA |

**Supplementary Table S1**. Studies meeting the meta-analysis inclusion criteria, and population characteristics. ARA: anti RNA polymerase III antibodies, DcSSc: diffuse cutaneous subset, ILD: interstitial lung disease, PH: pulmonary hypertension, SRC: scleroderma renal crisis.

**
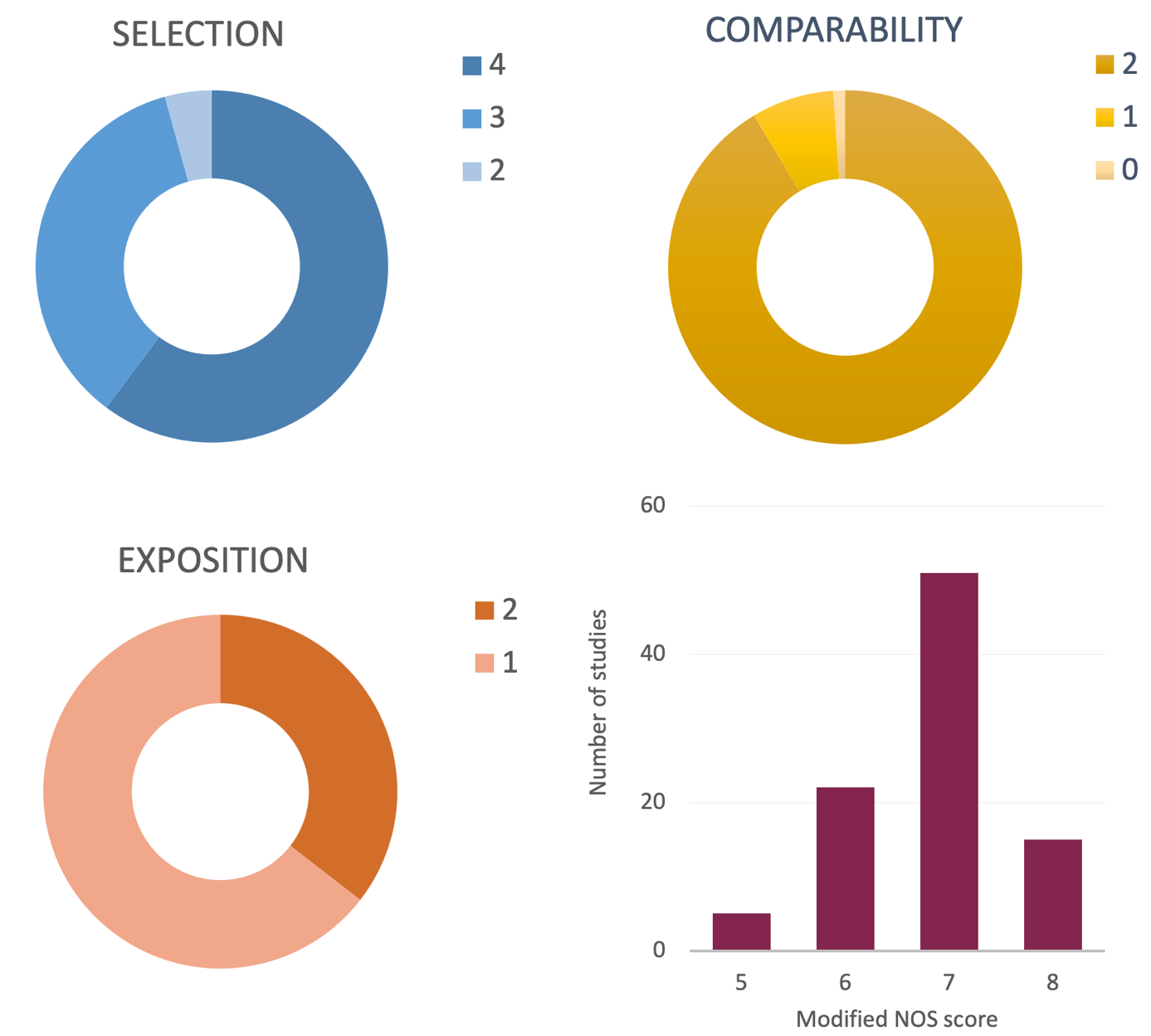
**

**Supplementary Figure S1.** Results of the methodologic assessment using the Newcastle-Ottawa Quality Assessment Scale (NOS) showing the scoring repartition in each of the three categories for the studies included in the meta-analysis, and histogram showing the number of studies per modified total NOS score (out of 8).


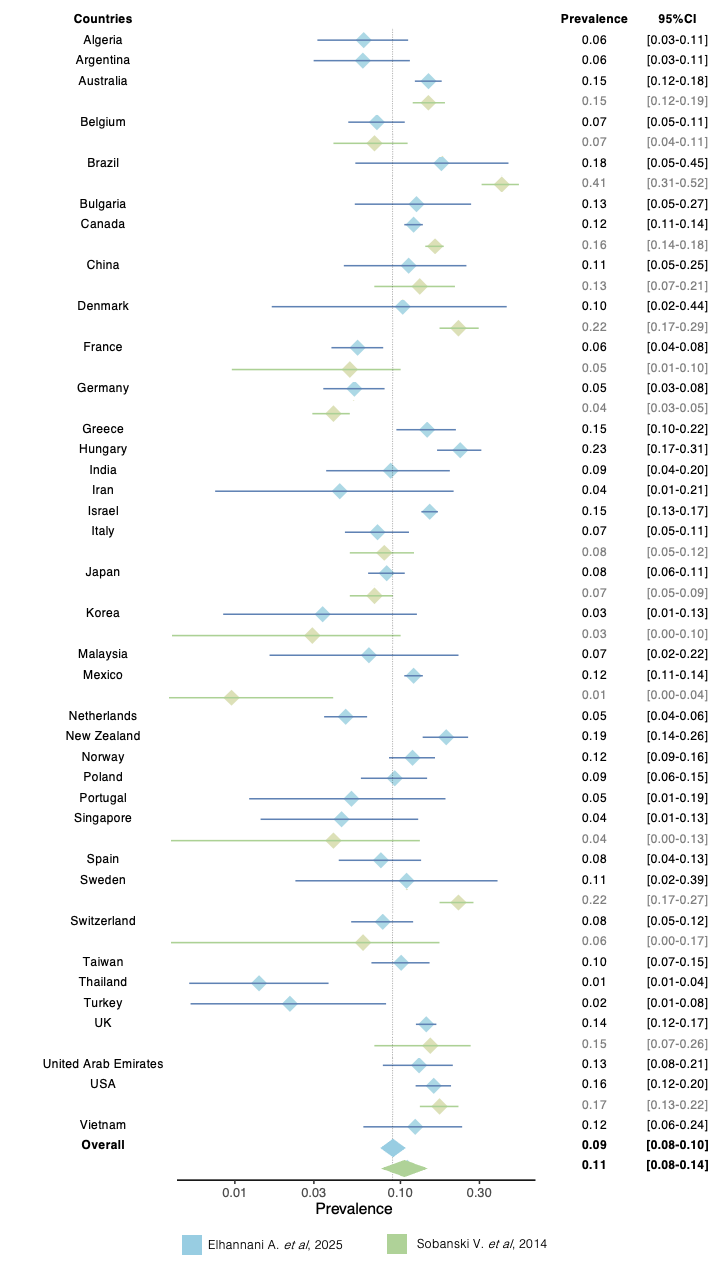


**Supplementary Figure S2**. Forest plot of ARA prevalence for each country included in the meta-analysis and comparison with the previous study on ARA from our group. Diamonds represents the pooled prevalence for each country. Lines represent the 95% confidence interval.

| **Variables** | ***p* association** | ***p* residual heterogeneity** |
| --- | --- | --- |
| Publication date | 0.22 | NA |
| Method of detection | **0.04** | **< 0.001** |
| Country | **0.006** | **< 0.001** |
| Continent | **< 0.001** | **< 0.001** |
| Age of cohort | 0.70 | NA |
| Female sex | 0.43 | NA |
| Skin involvement | 0.23 | NA |
| Disease duration | 0.37 | NA |
| Interstitial lung disease | 0.77 | NA |
| Pulmonary hypertension | 0.25 | NA |
| Digital ulcerations | **0.01** | **< 0.001** |
| Scleroderma renal crisis | 0.13 | NA |
| Anti-centromere antibodies | 0.29 | NA |
| Anti topoisomerase antibodies | 0.13 | NA |

**Supplementary Table S2.** Meta-regression analysis of the association between patients’ characteristics in included studies and ARA prevalence. NA: not applicable. Statistically significant associations are represented in bold.

| **Variables** | ***p* association** | ***p* residual heterogeneity** |
| --- | --- | --- |
| Country x method of detection | **< 0.0001** | **< 0.0001** |
| Method of detection x continent | **< 0.0001** | **< 0.0001** |
| Method of detection x digital ulcers | 0.14 | **< 0.0001** |
| Country x digital ulcers | 0.20 | **< 0.0001** |
| Continent x digital ulcers | **0.004** | **< 0.0001** |
| Country, method of detection x digital ulcers | **0.05** | **< 0.0001** |

**Supplementary Table S3.** Multiple meta-regression analysis of the association between patients’ characteristics in included studies and ARA prevalence. NA: not applicable. Statistically significant associations are represented in bold.

| **Variables** | ***p* association** | ***p* residual heterogeneity** |
| --- | --- | --- |
| ACA x ATA | **0.003** | **< 0.0001** |
| ATA x continent | **0.005** | **< 0.0001** |
| ACA x continent | **0.02** | **< 0.0001** |
| Disease duration x continent | **0.04** | **< 0.0001** |
| Disease duration x ACA | **0.006** | **< 0.0001** |
| Disease duration x ATA | **0.003** | **< 0.0001** |
| Disease duration, continent, ATA x ACA | **0.01** | **0.0009** |

**Supplementary Table S4.** Multiple meta-regression analysis of the association between characteristics of included studies and ARA association to cutaneous subset. NA: not applicable, ACA: anti-centromere antibodies, ATA: anti-topoisomerase I antibodies. Statistically significant associations are represented in bold.


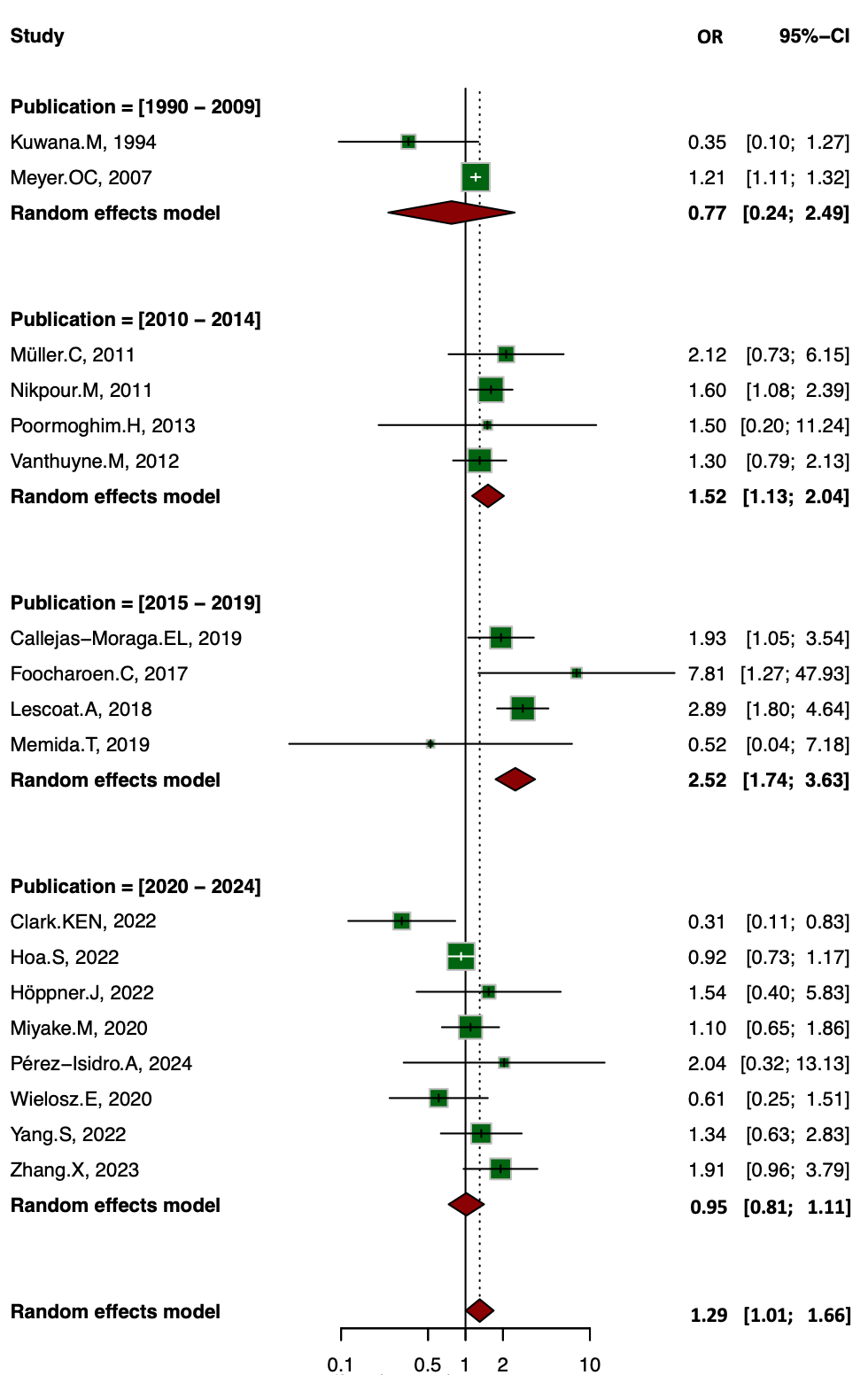


**Supplementary Figure S3**. Forest plot showing association between joint involvement and ARA in SSc patients, in all studies, according to the publication year. Each square represents an individual odds ratio, with the size of the square being proportional to the weight given to the study. Lines represent the 95% confidence interval. Diamonds represent the pooled odds ratio for each publication time frame.

| **Variables** | **OR [95% CI]** | ***p value*** | ***p het*** |
| --- | --- | --- | --- |
| Interstitial lung disease | **1.12 [1.02 ; 1.23]** | **0.02** | **NS** |
| Pulmonary hypertension | 1.00 [0.80 ; 1.26] | 0.97 | NS |
| Joint involvement | **1.36 [1.03 ; 1.80]** | **0.03** | **< 0.001** |
| DcSSc | **2.18 [1.85 ; 2.56]** | **< 0.001** | **< 0.001** |
| Digital ulcerations | 1.09 [0.99 ; 1.20] | 0.09 | NS |
| Muscle involvement | 1.15 [0.88 ; 1.49] | 0.32 | NS |
| Scleroderma renal crisis | **7.93 [5.74 ; 10.95]** | **< 0.001** | **0.02** |
| Female sex | **0.64 [0.53 ; 0.79]** | **< 0.001** | **NS** |
| Heart involvement | 1.05 [0.82 ; 1.36] | 0.67 | NS |
| Raynaud phenomenon | **1.02 [1.01 ; 1.03]** | **0.001** | **NS** |
| Esophageal involvement | **1.16 [1.03 ; 1.31]** | **0.02** | **0.02** |
| GI involvement | 1.04 [0.78 ; 1.38] | 0.78 | NS |
| Telangiectasia | 0.97 [0.89 ; 1.06] | 0.54 | NS |
| Calcinosis | 1.07 [0.96 ; 1.22] | 0.23 | NS |
| Cancer | **1.86 [1.33 ; 2.59]** | **< 0.001** | **NS** |
| GAVE | **2.70 [1.52 ; 4.81]** | **< 0.001** | **NS** |
| PBC | NA | NA | NA |
| Overlap syndrome | 0.56 [0.23 ; 1.37] | 0.20 | **0.04** |

**Supplementary Table S5.** Sensitivity analysis of the association between ARA seropositivity and SSc features, using studies published between 2010 and 2024. NA: not applicable, NS: not significant, DcSSc: diffuse cutaneous systemic sclerosis, GI involvement: gastrointestinal involvement, GAVE: gastric antral vascular ectasia, PBC: primary biliary cholangitis. Statistically significant associations are represented in bold.

**Supplementary references**:

1. LeRoy EC, Black C, Fleischmajer R, Jablonska S, Krieg T, Medsger TA, et al. Scleroderma (systemic sclerosis): classification, subsets and pathogenesis. J Rheumatol. 1988 Feb;15(2):202–5.

2. Abbot S, McWilliams L, Spargo L, de Costa C, Ur-Rehman Z, Proudman S, et al. Scleroderma in Cairns: an epidemiological study. Intern Med J. 2020 Apr;50(4):445–52.

3. Aguila LA, da Silva HC, Medeiros-Ribeiro AC, Bunjes BG, Luppino-Assad AP, Sampaio-Barros PD. Is exposure to environmental factors associated with a characteristic clinical and laboratory profile in systemic sclerosis? A retrospective analysis. Rheumatol Int. 2021 Jun;41(6):1143–50.

4. Andréasson K, Saxne T, Bergknut C, Hesselstrand R, Englund M. Prevalence and incidence of systemic sclerosis in southern Sweden: population-based data with case ascertainment using the 1980 ARA criteria and the proposed ACR-EULAR classification criteria. Ann Rheum Dis. 2014 Oct;73(10):1788–92.

5. Bardoni A, Rossi P, Salvini R, Bobbio-Pallavicini F, Caporali R, Montecucco C. Autoantibodies to RNA-polymerases in Italian patients with systemic sclerosis. Clin Exp Rheumatol. 2003;21(3):301–6.

6. Bass RD, Moore DF, Steen VD. Prevalence and Characteristics of Patients With Systemic Sclerosis Fulfilling the 2019 EULAR/American College of Rheumatology Classification Criteria for Systemic Lupus Erythematosus. Arthritis Care Res. 2024 Mar;76(3):311–7.

7. Boonstra M, Mertens BJA, Bakker JA, Ninaber MK, Ajmone Marsan N, van der Helm-van Mil AHM, et al. To what extent do autoantibodies help to identify high-risk patients in systemic sclerosis? Clin Exp Rheumatol. 2018;36 Suppl 113(4):109–17.

8. Bragazzi NL, Watad A, Gizunterman A, McGonagle D, Mahagna H, Comaneshter D, et al. The burden of depression in systemic sclerosis patients: a nationwide population-based study. J Affect Disord. 2019 Jan 15;243:427–31.

9. Caetano J, Batista F, Amaral MC, Oliveira S, Alves JD. Acute hospitalization in a cohort of patients with systemic sclerosis: a 10-year retrospective cohort study. Rheumatol Int. 2022 Aug;42(8):1393–402.

10. Callejas-Moraga EL, Guillén-Del-Castillo A, Marín-Sánchez AM, Roca-Herrera M, Balada E, Tolosa-Vilella C, et al. Clinical features of systemic sclerosis patients with anti-RNA polymerase III antibody in a single centre in Spain. Clin Exp Rheumatol. 2019;37 Suppl 119(4):41–8.

11. Catano J, Guedon A, Riviere S, Carrat F, Mahevas T, Fain O, et al. [Cancers in systemic sclerosis : risk factors, impact on survival and literature review]. Rev Med Interne. 2019 Oct;40(10):637–44.

12. Cavazzana I, Ceribelli A, Taraborelli M, Fredi M, Norman G, Tincani A, et al. Primary biliary cirrhosis-related autoantibodies in a large cohort of italian patients with systemic sclerosis. J Rheumatol. 2011 Oct;38(10):2180–5.

13. Ceribelli A, Isailovic N, De Santis M, Generali E, Satoh M, Selmi C. Detection of anti-mitochondrial antibodies by immunoprecipitation in patients with systemic sclerosis. J Immunol Methods. 2018 Jan;452:1–5.

14. Chang M, Wang RJ, Yangco DT, Sharp GC, Komatireddy GR, Hoffman RW. Analysis of autoantibodies against RNA polymerases using immunoaffinity-purifed RNA polymerase I, II, and III antigen in an enzyme-linked immunosorbent assay. Clin Immunol Immunopathol. 1998 Oct;89(1):71–8.

15. Clark KEN, Campochiaro C, Host LV, Sari A, Harvey J, Denton CP, et al. Combinations of scleroderma hallmark autoantibodies associate with distinct clinical phenotypes. Sci Rep. 2022 Jul 2;12(1):11212.

16. Coffey CM, Radwan YA, Sandhu AS, Crowson CS, Bauer PR, Matteson EL, et al. Epidemiology and Trends in Survival of Systemic Sclerosis in Olmsted County (1980-2018): A Population-based Study. J Scleroderma Relat Disord. 2021 Oct;6(3):264–70.

17. Dave J, Mahajan S, Khadilkar P, Pradhan V. Cutaneous Features, Autoantibody Profile, and Nailfold Capillaroscopy of Systemic Sclerosis: A Study of 60 Cases. J Assoc Physicians India. 2022 Nov;70(11):11–2.

18. De Almeida Chaves S, Porel T, Mounié M, Alric L, Astudillo L, Huart A, et al. Sine scleroderma, limited cutaneous, and diffused cutaneous systemic sclerosis survival and predictors of mortality. Arthritis Res Ther. 2021 Dec 7;23(1):295.

19. Didier K, Giusti D, Le Jan S, Terryn C, Muller C, Pham BN, et al. Neutrophil Extracellular Traps Generation Relates with Early Stage and Vascular Complications in Systemic Sclerosis. J Clin Med. 2020 Jul 7;9(7):2136.

20. Do HTT, Nguyen TNT, Le DH, Kanekura T. Auto-antibody profile and clinical presentation of Vietnamese with systemic sclerosis. Australas J Dermatol. 2021 May;62(2):e301–3.

21. Emilie S, Goulvestre C, Bérezné A, Pagnoux C, Guillevin L, Mouthon L. Anti-RNA polymerase III antibodies are associated with scleroderma renal crisis in a French cohort. Scand J Rheumatol. 2011;40(5):404–6.

22. Foocharoen C, Watcharenwong P, Netwijitpan S, Mahakkanukrauh A, Suwannaroj S, Nanagara R. Relevance of clinical and autoantibody profiles in systemic sclerosis among Thais. Int J Rheum Dis. 2017 Oct;20(10):1572–81.

23. Furukawa T, Matsui K, Kitano M, Yokoyama Y, Sekiguchi M, Azuma N, et al. Relationship between YKL-40 and pulmonary arterial hypertension in systemic sclerosis. Mod Rheumatol. 2019 May;29(3):476–83.

24. Gargiulo M de LÁ, Perez N, Khoury M, Buhl M, Suárez L, Sarano J, et al. Anti-RNA polymerase III antibodies in systemic sclerosis: Multicentric study from Argentina. Reumatol Clin. 2022;18(6):368–73.

25. Genrinho I, Ferreira PL, Santiago T, Carones A, Mazeda C, Barcelos A, et al. Validation of the Portuguese Version of the Scleroderma Health Assessment Questionnaire. Int J Environ Res Public Health. 2023 Nov 14;20(22):7062.

26. Gindzienska-Sieskiewicz E, Distler O, Reszec J, Jordan S, Bielecki P, Sieskiewicz A, et al. Increased expression of the TNF superfamily member LIGHT/TNFSF14 and its receptors (HVEM and LTßR) in patients with systemic sclerosis. Rheumatol Oxf Engl. 2019 Mar 1;58(3):502–10.

27. González-Martín JJ, Novella-Navarro M, Calvo-Aranda E, Cabrera-Alarcón JL, Carrión O, Abdelkader A, et al. Endothelial dysfunction and subclinical atheromatosis in patients with systemic sclerosis. Clin Exp Rheumatol. 2020;38 Suppl 125(3):48–52.

28. Graf SW, Hakendorf P, Lester S, Patterson K, Walker JG, Smith MD, et al. South Australian Scleroderma Register: autoantibodies as predictive biomarkers of phenotype and outcome. Int J Rheum Dis. 2012 Feb;15(1):102–9.

29. Günther F, Straub RH, Hartung W, Luchner A, Fleck M, Ehrenstein B. Increased Serum Levels of soluble ST2 as a Predictor of Disease Progression in Systemic Sclerosis. Scand J Rheumatol. 2022 Jul;51(4):315–22.

30. Harvey GR, Butts S, Rands AL, Patel Y, McHugh NJ. Clinical and serological associations with anti-RNA polymerase antibodies in systemic sclerosis. Clin Exp Immunol. 1999 Aug;117(2):395–402.

31. Hesselstrand R, Scheja A, Shen GQ, Wiik A, Akesson A. The association of antinuclear antibodies with organ involvement and survival in systemic sclerosis. Rheumatol Oxf Engl. 2003 Apr;42(4):534–40.

32. Hoa S, Lazizi S, Baron M, Wang M, Fritzler MJ, Hudson M, et al. Association between autoantibodies in systemic sclerosis and cancer in a national registry. Rheumatol Oxf Engl. 2022 Jul 6;61(7):2905–14.

33. Hoffmann-Vold AM, Midtvedt Ø, Tennøe AH, Garen T, Lund MB, Aaløkken TM, et al. Cardiopulmonary Disease Development in Anti-RNA Polymerase III-positive Systemic Sclerosis: Comparative Analyses from an Unselected, Prospective Patient Cohort. J Rheumatol. 2017 Apr;44(4):459–65.

34. Höppner J, Tabeling C, Casteleyn V, Kedor C, Windisch W, Burmester GR, et al. Comprehensive autoantibody profiles in systemic sclerosis: Clinical cluster analysis. Front Immunol. 2022;13:1045523.

35. Horimoto AMC, Matos ENN, Costa MR da, Takahashi F, Rezende MC, Kanomata LB, et al. Incidence and prevalence of systemic sclerosis in Campo Grande, State of Mato Grosso do Sul, Brazil. Rev Bras Reumatol. 2017;57(2):107–14.

36. Hübsch T, Mettler C, Poisnel E, Antoine C, Cambon A, Delarbre D, et al. [Autoimmune and inflammatory pathologies associated with systemic scleroderma: Clinical, serological and prognostic profiles. Bi-centric retrospective series in the PACA region]. Rev Med Interne. 2023 Aug;44(8):402–9.

37. Hysa E, Pizzorni C, Sammorì S, Gotelli E, Cere A, Schenone C, et al. Microvascular damage in autoimmune connective tissue diseases: a capillaroscopic analysis from 20 years of experience in a EULAR training and research referral centre for imaging. RMD Open. 2023 Jul;9(3):e003071.

38. Jacobsen S, Ullman S, Shen GQ, Wiik A, Halberg P. Influence of clinical features, serum antinuclear antibodies, and lung function on survival of patients with systemic sclerosis. J Rheumatol. 2001 Nov;28(11):2454–9.

39. Jung S, Gavriiloglou M, Séverac F, Haumesser L, Sayeh A, Chatelus E, et al. Influence of systemic sclerosis on periodontal health: A case-control study. J Clin Periodontol. 2023 Oct;50(10):1348–59.

40. Kang EH, Lee EB, Kim DJ, Im CH, Lee HJ, Song YW. Anti-RNA polymerase antibodies in Korean patients with systemic sclerosis and their association with clinical features. Clin Exp Rheumatol. 2005;23(5):731–2.

41. Karami J, Ghorban K, Kavosi H, Gharibdoost F, Dadmanesh M, Rouzbahani NH, et al. Evaluation of keratin 1 gene expression and its single nucleotide polymorphism (rs14024) in systemic sclerosis patients. Gene Rep. 2021 Dec 1;25:101404.

42. Korman BD, Marangoni RG, Hinchcliff M, Shah SJ, Carns M, Hoffmann A, et al. Brief Report: Association of Elevated Adipsin Levels With Pulmonary Arterial Hypertension in Systemic Sclerosis. Arthritis Rheumatol Hoboken NJ. 2017 Oct;69(10):2062–8.

43. Korsholm SS, Andersson DC, Knudsen JB, Dastmalchi M, Diederichsen ACP, Gerke O, et al. Myositis-specific autoantibodies and QTc changes by ECG in idiopathic inflammatory myopathies. Rheumatol Oxf Engl. 2022 Oct 6;61(10):4076–86.

44. Krzyszczak ME, Li Y, Ross SJ, Ceribelli A, Chan EKL, Bubb MR, et al. Gender and ethnicity differences in the prevalence of scleroderma-related autoantibodies. Clin Rheumatol. 2011 Oct;30(10):1333–9.

45. Kurteva E, Kalinova D, Velikova T, Tumangelova-Yuzeir K, Ivanova-Todorova E, Reshkova V, et al. A wide immunological profile in the diagnosis of progressive systemic sclerosis. 2016 Jan 1;24:35–51.

46. Kuwana M, Kaburaki J, Okano Y, Tojo T, Homma M. Clinical and prognostic associations based on serum antinuclear antibodies in Japanese patients with systemic sclerosis. Arthritis Rheum. 1994 Jan;37(1):75–83.

47. Lescoat A, Ballerie A, Belhomme N, Cazalets C, de Carlan M, Droitcourt C, et al. Synovial involvement assessed by power Doppler ultra-sonography in systemic sclerosis: results of a cross-sectional study. Rheumatol Oxf Engl. 2018 Nov 1;57(11):2012–21.

48. Liaskos C, Marou E, Simopoulou T, Barmakoudi M, Efthymiou G, Scheper T, et al. Disease-related autoantibody profile in patients with systemic sclerosis. Autoimmunity. 2017 Nov;50(7):414–21.

49. Lopez L, Barnetche T, Galli G, Seneschal J, Blanchard E, Shipley E, et al. Clinical and immunological features of patients with cancer-associated systemic sclerosis: An observational study. Joint Bone Spine. 2023 May;90(3):105555.

50. Low AHL, Wong S, Thumboo J, Ng SC, Lim JY, Ng X, et al. Evaluation of a new multi-parallel line immunoassay for systemic sclerosis-associated antibodies in an Asian population. Rheumatol Oxf Engl. 2012 Aug;51(8):1465–70.

51. Machhua S, Sharma SK, Kumar Y, Anand S, Handa S, Minz RW. Antinuclear antibody pattern and autoantibody profiling of systemic sclerosis patients in a tertiary referral center in North India. Pathol Int. 2022 May;72(5):283–92.

52. Majone F, Olivieri S, Cozzi F, Montaldi A, Tonello M, Visentin MS, et al. Increased apoptosis in circulating lymphocyte cultures of anti-RNA polymerase III positive patients with systemic sclerosis. Rheumatol Int. 2009 Jun;29(8):891–5.

53. Martel ME, Leurs A, Launay D, Behal H, Chepy A, Collet A, et al. Prevalence of anti-Ro52-kDa/SSA (TRIM21) antibodies and associated clinical phenotype in systemic sclerosis: Data from a French cohort, a systematic review and meta-analysis. Autoimmun Rev. 2024 May;23(5):103536.

54. Martins P, Dourado E, Fonseca JE, Cordeiro I, Romão V, Resende C. Ten years of a systemic sclerosis clinic in a tertiary referral centre - insights and future directions. Acta Reumatol Port. 2021;46(3):257–65.

55. Memida T, Matsuda S, Kajiya M, Mizuno N, Ouhara K, Fujita T, et al. Multiple External Root Resorption of Teeth as a New Manifestation of Systemic Sclerosis-A Cross-Sectional Study in Japan. J Clin Med. 2019 Oct 4;8(10):1628.

56. Meyer OC, Fertig N, Lucas M, Somogyi N, Medsger TA. Disease subsets, antinuclear antibody profile, and clinical features in 127 French and 247 US adult patients with systemic sclerosis. J Rheumatol. 2007 Jan;34(1):104–9.

57. Miyake M, Matsushita T, Takehara K, Hamaguchi Y. Clinical features of Japanese systemic sclerosis (SSc) patients negative for SSc-related autoantibodies: A single-center retrospective study. Int J Rheum Dis. 2020 Aug;23(9):1219–25.

58. Morozzi G, Bellisai F, Fineschi I, Scaccia F, Pucci G, Simpatico A, et al. Prevalence of anti-histone antibodies, their clinical significance and correlation with other autoantibodies in a cohort of Italian scleroderma patients. Auto- Immun Highlights. 2011 May;2(1):29–33.

59. Motegi SI, Toki S, Yamada K, Uchiyama A, Ishikawa O. Demographic and clinical features of systemic sclerosis patients with anti-RNA polymerase III antibodies. J Dermatol. 2015 Feb;42(2):189–92.

60. Müller C de S, Paiva EDS, Azevedo VF, Radominski SC, Lima Filho JHC. Autoantibody profile and clinical correlation in a group of patients with systemic sclerosis in southern Brazil. Rev Bras Reumatol. 2011;51(4):314–8, 323–4.

61. Nagy G, Minier T, Varjú C, Faludi R, Kovács KT, Lóránd V, et al. The presence of small joint contractures is a risk factor for survival in 439 patients with systemic sclerosis. Clin Exp Rheumatol. 2017;35 Suppl 106(4):61–70.

62. Namas R, Elarabi M, Khan S, Mubashir A, Memisoglu E, El-Kaissi M, et al. Comprehensive description of the prevalence, serological and clinical characteristics, and visceral involvement of systemic sclerosis (scleroderma) in a large cohort from the United Arab Emirates Systemic Sclerosis Registry. J Scleroderma Relat Disord. 2023 Jun;8(2):137–50.

63. Nguyen B, Mayes MD, Arnett FC, del Junco D, Reveille JD, Gonzalez EB, et al. HLA-DRB1*0407 and *1304 are risk factors for scleroderma renal crisis. Arthritis Rheum. 2011 Feb;63(2):530–4.

64. Nikpour M, Hissaria P, Byron J, Sahhar J, Micallef M, Paspaliaris W, et al. Prevalence, correlates and clinical usefulness of antibodies to RNA polymerase III in systemic sclerosis: a cross-sectional analysis of data from an Australian cohort. Arthritis Res Ther. 2011;13(6):R211.

65. Norman GL, Bialek A, Encabo S, Butkiewicz B, Wiechowska-Kozlowska A, Brzosko M, et al. Is prevalence of PBC underestimated in patients with systemic sclerosis? Dig Liver Dis Off J Ital Soc Gastroenterol Ital Assoc Study Liver. 2009 Oct;41(10):762–4.

66. Nowakowska-Płaza A, Wroński J, Werońska-Tatara J, Foryś A, Kraska A, Wisłowska M. Heart disease in the course of systemic sclerosis - an observational study. Reumatologia. 2022;60(5):318–25.

67. Oller-Rodríguez JE, Vicens Bernabeu E, Gonzalez-Mazarío R, Grau García E, Ortiz Sanjuan FM, Román Ivorra JA. Utility of cytokines CXCL4, CXCL8 and GDF15 as biomarkers in systemic sclerosis. Med Clin (Barc). 2022 Oct 28;159(8):359–65.

68. Osthoff M, Jaeger VK, Heijnen IAFM, Trendelenburg M, Jordan S, Distler O, et al. Role of lectin pathway complement proteins and genetic variants in organ damage and disease severity of systemic sclerosis: a cross-sectional study. Arthritis Res Ther. 2019 Mar 18;21(1):76.

69. Pérez-Isidro A, Lledó-Ibáñez GM, de Moner N, Torradeflot M, Martínez MJ, Espinosa G, et al. Could the IgA isotype provide additional information in systemic sclerosis patients? A retrospective study entailing IgA isotyping in a Mediterranean systemic sclerosis cohort. Clin Exp Rheumatol. 2024 Aug;42(8):1556–63.

70. Poormoghim H, Moghadam AS, Moradi-Lakeh M, Jafarzadeh M, Asadifar B, Ghelman M, et al. Systemic sclerosis: demographic, clinical and serological features in 100 Iranian patients. Rheumatol Int. 2013 Aug;33(8):1943–50.

71. Potjewijd J, Tobal R, Silvertand D, Gietema HA, Damoiseaux JGMC, van Paassen P. Favorable long term effects of intensified immunosuppression combined with therapeutic plasma exchange in patients with early-onset progressive systemic sclerosis-related interstitial lung disease. J Transl Autoimmun. 2022;5:100174.

72. Ramahi A, Lescoat A, Roofeh D, Nagaraja V, Namas R, Huang S, et al. Risk factors for lung function decline in systemic sclerosis-associated interstitial lung disease in a large single-centre cohort. Rheumatol Oxf Engl. 2023 Jul 5;62(7):2501–9.

73. Rees MS, Frampton C, White DHN, Solanki KK. Increased malignancies in our Waikato cohort of patients with systemic sclerosis. Int J Rheum Dis. 2021 Apr;24(4):555–61.

74. Richardson C, Perin J, Zeger S, Wigley FM, Hummers LK, Casciola-Rosen L, et al. Cumulative disease damage and anti-PM/Scl antibodies are associated with a heavy burden of calcinosis in systemic sclerosis. Rheumatol Oxf Engl. 2023 Nov 2;62(11):3636–43.

75. Serling-Boyd N, Chung MPS, Li S, Becker L, Fernandez-Becker N, Clarke J, et al. Gastric antral vascular ectasia in systemic sclerosis: Association with anti-RNA polymerase III and negative anti-nuclear antibodies. Semin Arthritis Rheum. 2020 Oct;50(5):938–42.

76. Sheikh MB, Naqati SM, Ahmad M, Soharwardy MY, Rather BA, Qayoom S, et al. Clinical and Serological Profile of Systemic Sclerosis Patients in a Tertiary Care Center in Kashmir, North India. Indian J Rheumatol. 2023 Dec;18(4):276.

77. Smeets RL, Kersten BE, Joosten I, Kaffa C, Alkema W, Koenen HJPM, et al. Diagnostic profiles for precision medicine in systemic sclerosis; stepping forward from single biomarkers towards pathophysiological panels. Autoimmun Rev. 2020 May;19(5):102515.

78. Sujau I, Ng CT, Sthaneshwar P, Sockalingam S, Cheah TE, Yahya F, et al. Clinical and autoantibody profile in systemic sclerosis: baseline characteristics from a West Malaysian cohort. Int J Rheum Dis. 2015 May;18(4):459–65.

79. Tahiat A, Allam I, Abdessemed A, Mellal Y, Nebbab R, Ladjouze-Rezig A, et al. Autoantibody profile in a cohort of Algerian patients with systemic sclerosis. Ann Biol Clin (Paris). 2020 Apr 1;78(2):126–33.

80. Tanaka H, Okada Y, Nakayamada S, Miyazaki Y, Sonehara K, Namba S, et al. Extracting immunological and clinical heterogeneity across autoimmune rheumatic diseases by cohort-wide immunophenotyping. Ann Rheum Dis. 2024 Jan 11;83(2):242–52.

81. Terras S, Hartenstein H, Höxtermann S, Gambichler T, Kreuter A. RNA polymerase III autoantibodies may indicate renal and more severe skin involvement in systemic sclerosis. Int J Dermatol. 2016 Aug;55(8):882–5.

82. Tsuji H, Kuramoto N, Sasai T, Shirakashi M, Onizawa H, Kitagori K, et al. Autoantibody profiles associated with morbidity and mortality in scleroderma renal crisis. Rheumatol Oxf Engl. 2022 Oct 6;61(10):4130–5.

83. Vanthuyne M, Smith V, De Langhe E, Van Praet J, Arat S, Depresseux G, et al. The Belgian Systemic Sclerosis Cohort: correlations between disease severity scores, cutaneous subsets, and autoantibody profile. J Rheumatol. 2012 Nov;39(11):2127–33.

84. Vemulapalli S, Cohen L, Hsu V. Prevalence and risk factors for left ventricular diastolic dysfunction in a scleroderma cohort. Scand J Rheumatol. 2017 Jul;46(4):281–7.

85. Villalta D, Imbastaro T, Di Giovanni S, Lauriti C, Gabini M, Turi MC, et al. Diagnostic accuracy and predictive value of extended autoantibody profile in systemic sclerosis. Autoimmun Rev. 2012 Dec;12(2):114–20.

86. Watanabe T, Ototake Y, Akita A, Suzuki M, Kanaoka M, Tamura J, et al. Clinical features of patients with systemic sclerosis positive for anti-SS-A antibody: a cohort study of 156 patients. Arthritis Res Ther. 2024 May 3;26(1):93.

87. Wielosz E, Dryglewska M, Majdan M. Clinical consequences of the presence of anti-RNA Pol III antibodies in systemic sclerosis. Postepy Dermatol Alergol. 2020 Dec;37(6):909–14.

88. Yamaoka T, Ogawa F, Muroi E, Hara T, Komura K, Iwata Y, et al. Autoantibody against a protease domain of caspase-8 in patients with systemic sclerosis. Clin Exp Rheumatol. 2008;26(6):998–1004.

89. Yang S, Liang M, Chen C, Ye W, Zhu X, Xue Y, et al. Clinical correlations with disease-associated auto-antibodies in a Chinese cohort with systemic sclerosis. Chin Med J (Engl). 2022 Aug 5;135(15):1878–80.

90. Yayla ME, İlgen U, Düzgün N. An analysis of the relationship between autoantibodies and clinical findings in patients with systemic sclerosis. Turk J Med Sci. 2018 Feb 23;48(1):10–5.

91. Yen TH, Chen JP, Hsieh TY, Hung WT, Lai KL, Hsieh CW, et al. The diagnostic and prognostic value of a line immunoblot assay in Taiwanese patients with systemic sclerosis. Clin Chim Acta Int J Clin Chem. 2023 Jul 1;547:117457.

92. Żebryk P, Przymuszała P, Nowak JK, Piorunek T, Mularek-Kubzdela T, Puszczewicz M. Autoantibodies and Clinical Correlations in Polish Systemic Sclerosis Patients: A Cross-Sectional Study. J Clin Med. 2023 Jan 13;12(2):657.

93. Zhang X, Zhang H, Zhao J, Li Y, Wang H, Li C. Diagnostic accuracy and predictive value of autoantibody profiles in patients with systemic sclerosis: a single-center study. Clin Rheumatol. 2023 May;42(5):1297–306.

94. Ziswiler HR, Urech R, Balmer J, Ostensen M, Mierau R, Villiger PM. Clinical diagnosis compared to classification criteria in in a cohort of 54 patients with systemic sclerosis and associated disorders. Swiss Med Wkly. 2007 Oct 20;137(41–42):586–90.
